# Real-Time Measurement of a Weak Interaction of a Transcription Factor Motif with a Protein Hub at Single-Molecule Precision

**DOI:** 10.1021/acsnano.4c04857

**Published:** 2024-07-25

**Authors:** Lauren A. Mayse, Yazheng Wang, Mohammad Ahmad, Liviu Movileanu

**Affiliations:** † Department of Physics, Syracuse University, 201 Physics Building, Syracuse, New York 13244, United States; ‡ Department of Biomedical and Chemical Engineering, Syracuse University, 329 Link Hall, Syracuse, New York 13244, United States; § Department of Biology, Syracuse University, 114 Life Sciences Complex, Syracuse, New York 13244, United States; ∥ The BioInspired Institute, Syracuse University, Syracuse, New York 13244, United States

**Keywords:** protein dynamics, single-molecule electrophysiology, protein engineering, protein−protein interactions, biosensors, nanopores

## Abstract

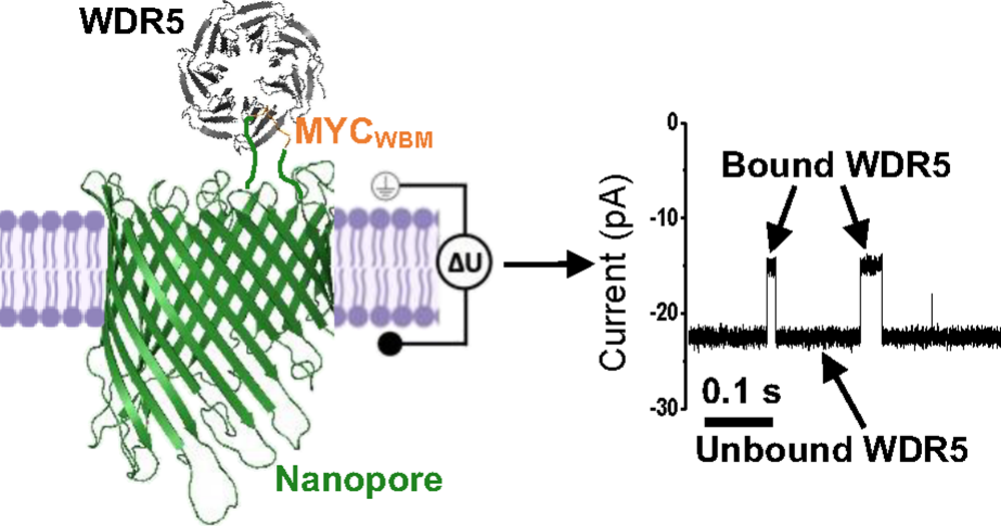

Transcription factors often interact with other protein cofactors, regulating gene expression. Direct detection of these brief events using existing technologies remains challenging due to their transient nature. In addition, intrinsically disordered domains, intranuclear location, and lack of cofactor-dependent active sites of transcription factors further complicate the quantitative analysis of these critical processes. Here, we create a genetically encoded label-free sensor to identify the interaction between a motif of the MYC transcription factor, a primary cancer driver, and WDR5, a chromatin-associated protein hub. Using an engineered nanopore equipped with this motif, WDR5 is probed through reversible captures and releases in a one-by-one and time-resolved fashion. Our single-molecule kinetic measurements indicate a weak-affinity interaction arising from a relatively slow complex association and a fast dissociation of WDR5 from the tethered motif. Further, we validate this subtle interaction by determinations in an ensemble using single nanodisc-wrapped nanopores immobilized on a biolayer interferometry sensor. This study also provides the proof-of-concept for a sensor that reveals unique recognition signatures of different protein binding sites. Our foundational work may be further developed to produce sensing elements for analytical proteomics and cancer nanomedicine.

Transcription factors (TFs) are essential members of the human proteome with the primary mission of regulating gene expression.^1^ Their interactions with deoxyribonucleic acid (DNA) regions and other protein coregulators mediate this critical function.^2−4^ In addition, TFs control disease development, so their interfaces with protein cofactors are potentially pursued as essential therapeutic targets. For example, c-myelocytomatosis (MYC), an oncoprotein TF, uses the druggable WD repeat-containing protein 5 (WDR5) as a cofactor.^5−8^ MYC is overexpressed in 70% of human cancers and controls genes that influence cell development and apoptosis.^9−13^ Experimental evidence indicated that MYC interacts with WDR5,^7,14^ utilizing one of its intrinsically disordered regions and the WDR5 binding motif (WBM) site.^15^ For simplicity, this binding region of MYC is called MYC_WBM_ (Figure 1a,b and Table S1). The MYC_WBM_–WDR5 interaction is expectedly weak, like other TF-protein cofactor interactions, yet it is required for tumorigenesis.^6^ Due to the complex interactome of WDR5,^16^ analyzing the nature of this interaction using prevailing technologies in the bulk phase is difficult. This technical shortcoming also holds for many other weak protein–protein interactions (PPIs) in cell signaling pathways under physiological and disease-like conditions. These weak PPIs are typically translated in rare or shortly lived binding events, whose duration is usually below the resolution of existing approaches.^17−19^

**Figure 1:**
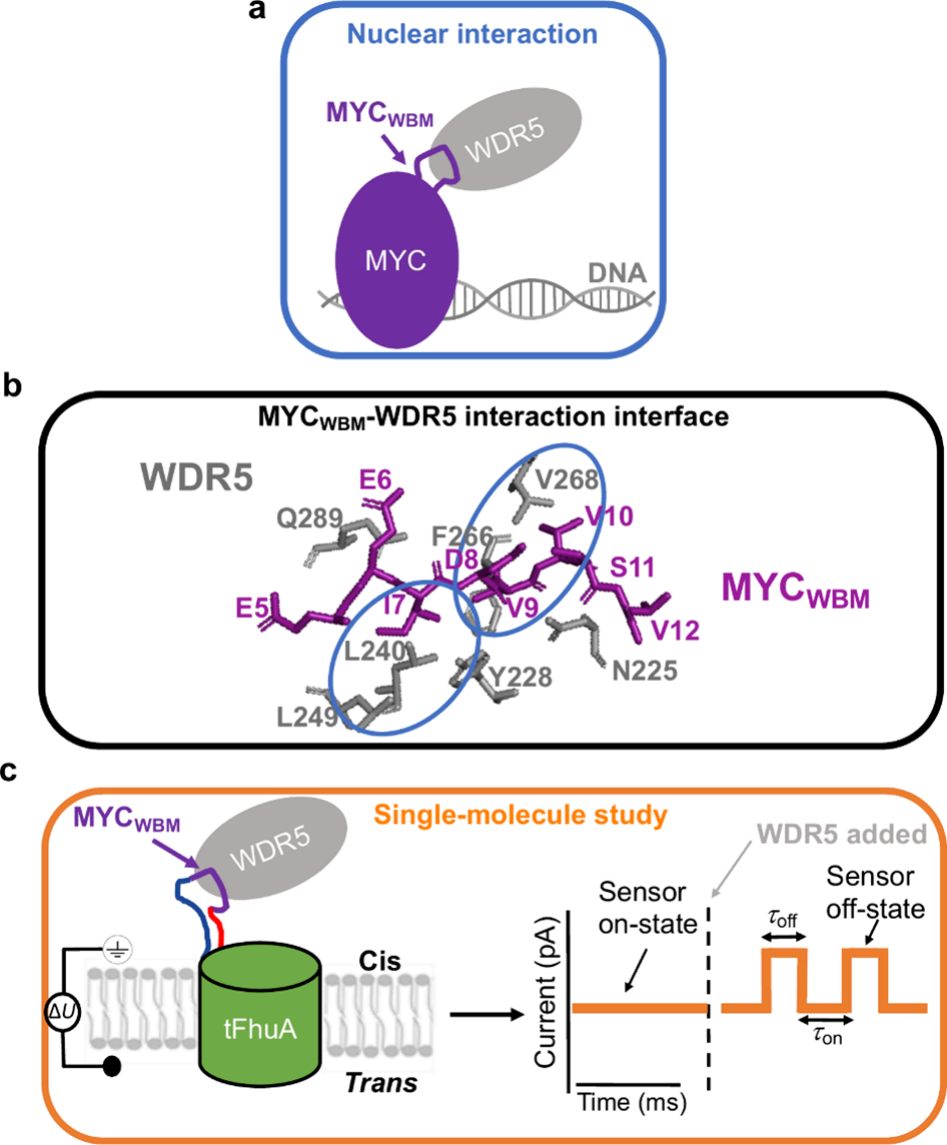
Experimental design for probing the MYC_WBM_–WDR5 interaction. (a) Cartoon of MYC_WBM_, a peptide ligand from the oncoprotein c-MYC, interacting with WDR5, a chromatin-associated protein hub, and dsDNA. (b) Critical residues of the MYC_WBM_–WDR5 interaction (PDB 4Y7R).^6^ The essential interaction residues for MYC_WBM_ (EEIDVVSV) are marked in purple. The crucial interaction residues for WDR5 are denoted in gray. Areas of hydrophobic contacts at the MYC_WBM_–WDR5 interaction interface are marked in blue ellipses. (c) On the left side, the cartoon shows the MYC_WBM_tFhuA nanopore sensor, including the tether (blue) and adaptor (red), reconstituted into a lipid bilayer, mimicking the MYC_WBM_–WDR5 interaction. On the right side is a schematical representation of how the electrical signature varies when a sensor detects the targeted interaction. The “on” and “off” states indicate when WDR5 is unbound and bound to MYC_WBM_, respectively. τ_on_ and τ_off_ denote the durations for unbound and bound WDR5, respectively.

We overcome these technical difficulties by employing a genetically encoded nanopore sensor and the resistive-pulse technique.^20^ Nanopore technologies^21−26^ can probe protein dynamics in a broad dynamic range due to a wide time bandwidth.^27^ This strategy creates customizable biosensors to identify protein interactions in a real-time and label-free setting.^28−31^

The capability to detect binding events^32−34^ with a substantially large time bandwidth, without the confinement of the nanopore interior, and at adjustable protein concentrations is critical when evaluating the MYC_WBM_–WDR5 interaction. In particular, the main benefits of using biological nanopores include their amenability to (i) structural and compositional alterations with atomic precision,^25^ (ii) integration into nanofluidic devices for parallel recordings technologies,^35^ and (iii) detection in complex biofluids and mixtures of proteins.^36−44^ These technological advantages enable a broad range of applications in protein analytics, such as enzymology,^45,46^ cotranslocational unfolding,^47,48^ post-translational modifications,^49−54^ mechanical stability,^23,34^ and peptide and protein fingerprinting.^55,56^ In recent years, engineered protein nanopores have also been actively utilized in the challenging aspects of single-molecule protein sequencing.^57−59^

In this study, we design, create, and validate a nanopore sensor using a monomeric 22-stranded β-barrel protein pore named tFhuA.^39^ We fuse the 13-residue MYC_WBM_ peptide ligand (QEDEEEIDVVSVE) to the N terminus of tFhuA via a flexible spacer. In addition, a peptide adaptor was covalently attached to this single-polypeptide chain protein nanopore to create the MYC_WBM_tFhuA sensor (Experimental Section, Figure 1c). Here, we employ this genetically encoded sensor to interrogate the interaction between the binding fragment of the oncoprotein MYC, MYC_WBM_, and 334-residue WDR5, a transcriptional coregulator. The role of the adaptor is to facilitate the detection of individual captures of WDR5 by the tethered MYC_WBM_ peptide ligand. Our event analysis confirms a weak-affinity MYC_WBM_–WDR5 interaction with the equilibrium dissociation constant (*K*_D_) in the micromolar range. This outcome results from a moderate-to-slow association and a fast dissociation of the complex. A systematic series of additional experiments reveals that the MYC_WBM_–WDR5 complex is, to some extent, electrostatically enriched yet stabilized by the two hydrophobic pockets of the WBM site. While this interaction has an intricate balance of electrostatic and hydrophobic contributions, our nanopore sensor detects uniform WBM-mediated WDR5 captures by the attached MYC_WBM_ peptide ligand. This finding contrasts the multimodal protein recognition of WDR5 through a deep cavity site via diverse subpopulations of binding events. Hence, this nanopore sensing strategy uniquely generates the ability to discriminate distinct protein recognition signatures for different binding sites of the same protein hub. More broadly, our sensor probes the binding interface of a protein cofactor with a TF binding ligand in a time-resolved fashion and with a single-molecule precision.

## Results and Discussion

### Evaluation of the weak MYC_WBM_–WDR5 interaction

**Figure 2:**
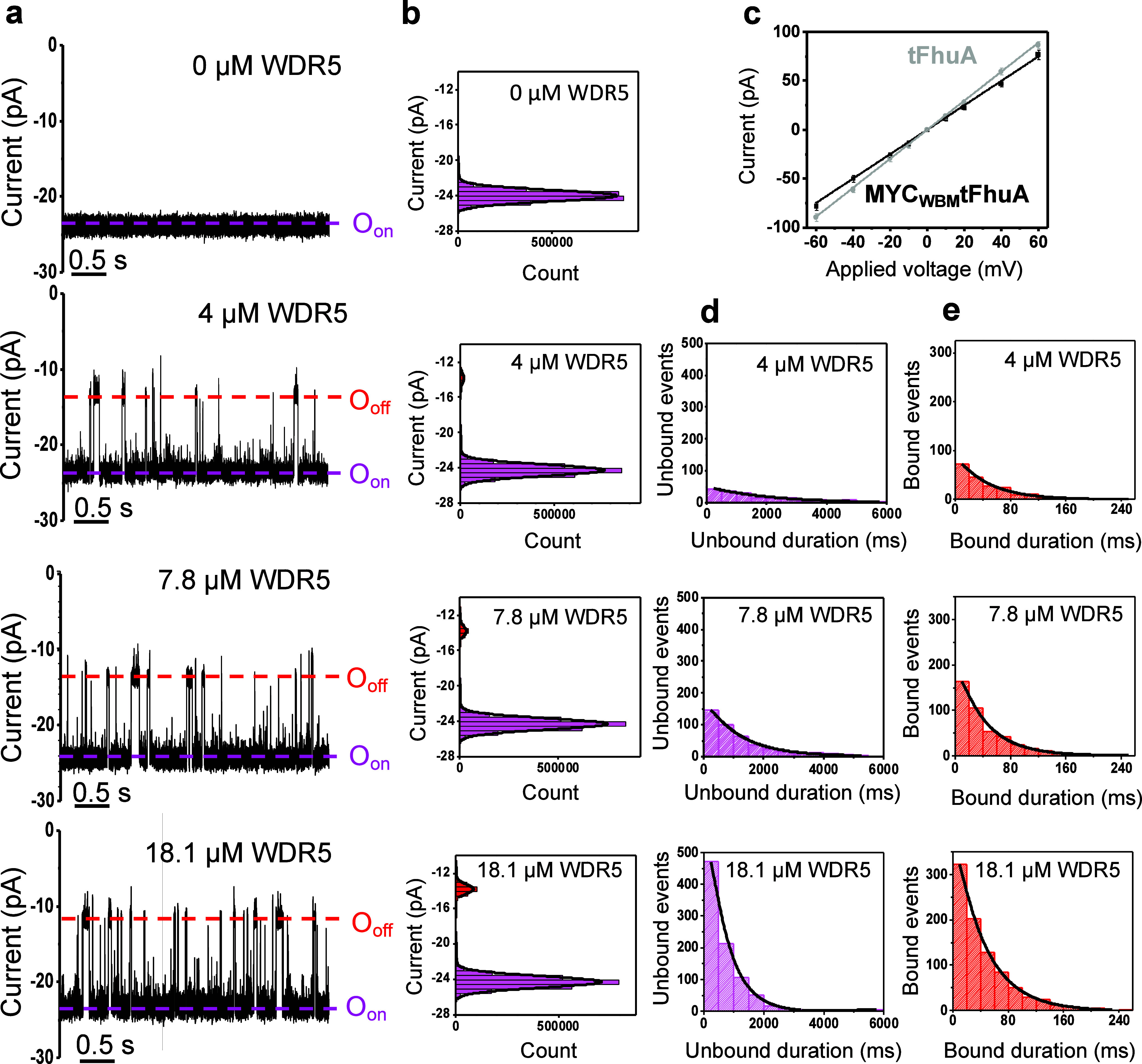
Single-molecule detection of the MYC_WBM_–WDR5 interaction. (a) Single-channel electrical recording of MYC_WBM_tFhuA at various WDR5 concentrations ([WDR5]). *O*_on_ and *O*_off_ indicate the unbound and bound substates, respectively. All single-channel electrical signatures were replicated in at least *n* = 4 independent single-molecule reconstitution experiments. Electrical traces were low-pass filtered at 1 kHz using an 8-pole Bessel filter. The applied transmembrane potential was −20 mV. All recordings were conducted in 300 mM KCl, 20 mM Tris-HCl, and pH 7.5. (b) All-point current amplitude histograms of the MYC_WBM_tFhuA nanopore sensor at various [WDR5] values. (c) *I*/*V* curves of the unmodified tFhuA and MYC_WBM_tFhuA nanopores. These plots indicate the open-state current as a function of the transmembrane potential for the unmodified tFhuA nanopore (gray circles) and MYC_WBM_tFhuA sensor (black squares). Data points represent mean ± s.d. from *n* = 4 independent nanopore experiments. (d) Standard event histograms of unbound durations (τ_on_) at various [WDR5] values. The τ_on_ values (mean ± s.e.m.) were 1,560 ± 20 ms (the number of events: *N* = 201), 879 ± 22 ms (*N* = 457), and 419 ± 7 ms (*N* = 889) in the presence of 4, 7.8, and 18.1 μM WDR5, respectively. (e) Standard event histograms of bound durations (τ_off_). The τ_off_ values (mean ± s.e.m.) were 40 ± 4 ms (the number of events: *N* = 203), 37 ± 3 ms (*N* = 436), and 39 ± 4 ms (*N* = 875) in the presence of 4, 7.8, and 18.1 μM WDR5, respectively.

MYC_WBM_tFhuA exhibited a quiet open-state current of −24 ± 2 pA at a transmembrane potential of −20 mV and in a solution containing 300 mM KCl, 20 mM Tris-HCl, 1 mM TCEP, and pH 7.5 (*n* = 8 independently reconstituted nanopores; Figure 2a,b, top panels; Table S2). Notably, this unitary current was lower than that corresponding to the unmodified tFhuA nanopore (−30 ± 3 pA, *n* = 8), likely due to the MYC_WBM_ peptide ligand hanging over the *cis* entrance of the pore (Figure 2c, Figure S1). When a single MYC_WBM_tFhuA was reconstituted into a membrane, WDR5 added to the *cis* side produced transient current blockades, whose amplitude was independent of the WDR5 concentration, [WDR5] (Figure 2a,b, panels on lines 2–4; Table S3). Here, *O*_on_ denotes the open substate or the WDR5-unbound substate, named the unbound substate. *O*_off_ indicates the WDR5-bound substate, called the bound substate.

No significant current blockades were detected when WDR5 was added to the chamber with an unmodified tFhuA-containing membrane (Figure S1). Using this negative-control experiment, we show that WDR5-produced current transitions were not caused by nonspecific interactions between WDR5 and the *cis* entrance on the pore. In addition, we tested an adaptor-free variant of MYC_WBM_tFhuA and found no current blockades in the presence of WDR5 (Figure S2). This control experiment indicates the necessity of the peptide adaptor for sensing the MYC_WBM_–WDR5 interaction outside the pore lumen. The maximum likelihood method^60^ and logarithm likelihood ratio (LLR)^61^ tests were employed for all fittings of event duration histograms to determine their best model for the probability distribution function. At a confidence level *C* = 0.95, a single-exponential fit was the best model for the unbound (Figure 2d) and bound (Figure 2e) durations of the MYC_WBM_–WDR5 interaction.

**Figure 3:**
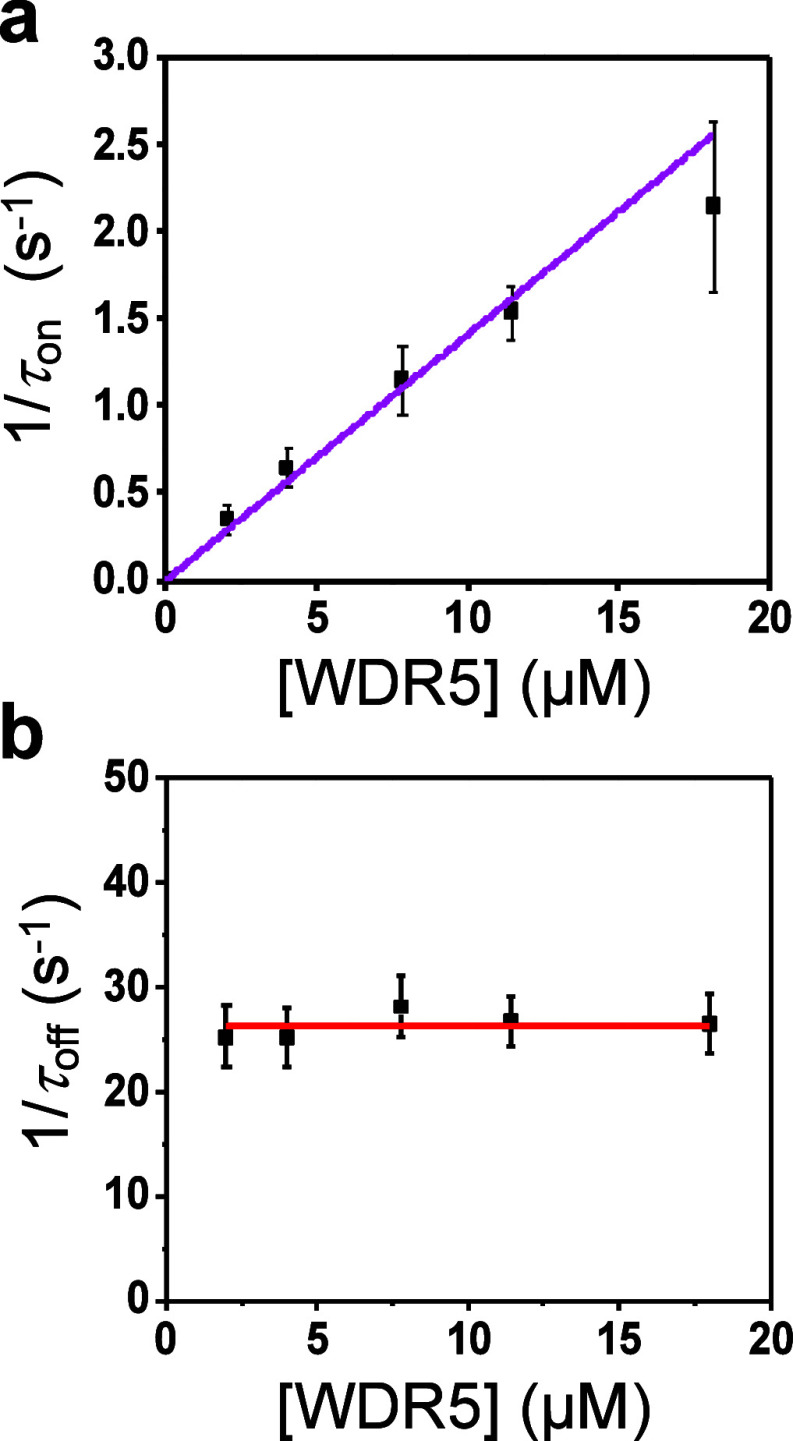
Dose–response curves of the event frequency and dissociation rate constant for the MYC_WBM_–WDR5 interaction. (a) Event frequency is in the form of 1*/*τ_on_ versus [WDR5]. (b) Dissociation rate constant, *k*_off_, in the form of 1*/*τ_off_ versus [WDR5]. The data represents mean ± s.d. obtained from *n* = 4 distinct experiments.

The frequency of the bound events increased at elevated [WDR5] values (Figure 2a). This frequency amplification corresponded to a decrease in the unbound duration, τ_on_, but with no change in the bound duration, τ_off_ (Table S4). Because τ_off_ was independent of [WDR5], a unimolecular dissociation mechanism of the MYC_WBM_–WDR5 complex was observed. We also noted that the frequency of binding events increased linearly and at a 1:1 ratio with the [WDR5] value (Figure 3a), confirming a bimolecular association process of the MYC_WBM_–WDR5 interaction.

The association rate constant, *k*_on,_ for this interaction was determined through the slope of the linear fit of the event frequency, *f* (*f* = 1/τ_on_), versus [WDR5]. The *k*_on_ (mean ± s.e.m.) was (1.4 ± 0.1) × 10^5^ M^–1^ s^–1^ (Table S5). This is a relatively low value near the basal range for the diffusion-controlled regime of PPIs (e.g., 10^5^–10^6^ M^–1^ s^–1^).^62^ Other physical and environmental aspects matter in determining the *k*_on_, including the diffusion coefficient of the individual binding partners and potential biasing forces that either speed up or slow down the association process. In this study, MYC_WBM_ is a relatively short peptide ligand immobilized onto a lipid bilayer surface via a nanopore scaffold, a translational constraint that severely declines the *k*_on_. The MYC_WBM_–WDR5 interaction is also featured by short-range hydrophobic forces (see below) that usually have some impact on the association of the transient MYC_WBM_–WDR5 complex.^62^ In addition, the dissociation rate constant, *k*_off_, was determined as the *y*-intercept of the horizontal line fit of 1/τ_off_ with the vertical axis (Figure 3b). The *k*_off_ (mean ± s.e.m.) was 26 ± 1 s^–1^ (Table S6), which resulted from a brief bound duration of ∼38 ms.

### Qualitative and Quantitative Validations of the Weak-Affinity MYC_WBM_–WDR5 Interaction

The equilibrium dissociation constant, *K*_D_, provided insight into the strength of the MYC_WBM_–WDR5 interaction and was indirectly determined as *K*_D_ = *k*_off_ /*k*_on._ The *K*_D_ (mean ± s.d.) was 200 ± 21 μM (*n* = 10) (Table S7). This weak binding affinity resulted from a relatively high *k*_off_ value due to a brief bound duration and a relatively low *k*_on_ value. Then, we tested the same interaction using the functional reconstitution of MYC_WBM_tFhuA into nanodiscs (ND), which were subsequently immobilized onto a biolayer interferometry (BLI) sensor (Experimental Section). This nanodisc (ND)-integrated BLI approach (ND-BLI) was used to monitor the MYC_WBM_–WDR5 interaction in an ensemble. This way, the WDR5 protein recognition outside the pore lumen can be evaluated using an optical detection modality independent of the resistive-pulse technique. Hence, we selected ND-BLI as our validation route because it directly determines *k*_on_ and *k*_off_ using a real-time, label-free approach. However, a quantitative evaluation of the MYC_WBM_–WDR5 interaction was impossible due to the limited time resolution of BLI (e.g., very short-lived binding events).^63^ Yet, the ND-BLI qualitatively confirmed both a relatively slow physical association of the MYC_WBM_–WDR5 complex (e.g., a slow BLI response in the association phase) and a fast dissociation process (e.g., an abrupt decline in the BLI response in the dissociation phase) (Figure S3).

Experimental SectionFurthermore, this outcome motivated us to conduct steady-state fluorescence polarization (FP) anisotropy^64,65^ experiments to confirm this weak interaction (Experimental Section). When a short, fluorescein isothiocyanate (FITC)-labeled MYC_WBM_ peptide interacts with WDR5 (∼36.6 kDa),^66^ it induces a reduction in its tumbling rate, leading to an increased steady-state FP anisotropy. Consistent with our initial prediction, we observed a substantial rise in the steady-state FP anisotropy at higher [WDR5] values (Figure S4), confirming the specific interaction between the FITC-labeled MYC_WBM_ and WDR5. The calculated *K*_D_ value of the MYC_WBM_–WDR5 interaction using an equilibrium binding curve was 5.0 ± 0.9 μM. This value is in good accordance with prior FP anisotropy determinations of the MbIIIb-WDR5 complex by Thomas and co-workers, who acquired a *K*_D_ of ∼9.3 μM in 300 mM NaCl.^6^ Here, the sequence of MbIIIb is DEEEIDVVSV. It is also important to note that alterations in physicochemical conditions, such as the ionic strength and immobilization of one binding partner onto a surface, can substantially alter the binding affinity of the interacting molecules.^42^ Therefore, the significantly weaker binding interaction observed with the MYC_WBM_-containing nanopore sensor immobilized on a lipid bilayer compared to the value measured freely in solution using the steady-state FP spectroscopy can be attributed to these factors.

### Dependence of the MYC_WBM_–WDR5 Interaction on the Ionic Strength

The binding interface of the MYC_WBM_–WDR5 complex shows some stabilizing hydrophobic contacts and hydrogen bonds while also containing ion-pair interactions (Table S1).^6^ In addition, the negative charge of MYC_WBM_ (pI_MYCWBM_ = 3.25) and the positive charge of WDR5 (pI_WDR5_ = 8.27) suggest that long-range electrostatic forces may tune this complex’s association and dissociation kinetics. A significant advantage of tFhuA-based nanopores is their large conductance of ∼1.2 nS at 300 mM KCl,^39,40^ providing a high signal-to-noise ratio. Hence, these electrical recordings can be conducted at a very low salt concentration while maintaining the integrity of the monomeric β-barrel structure and pore-forming properties of the sensor.

**Figure 4:**
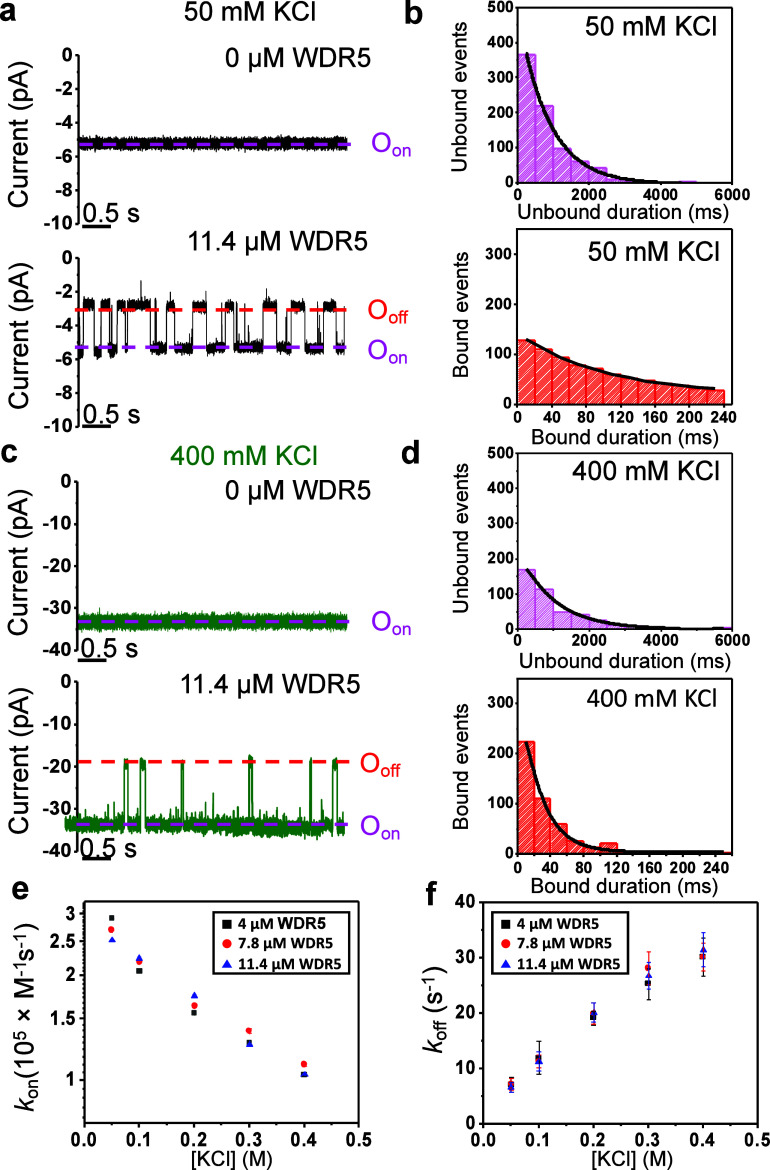
Alterations in the strength of the MYC_WBM_–WDR5 interaction through changes in the ionic strength. (a) Single-channel recording of MYC_WBM_tFhuA in 50 mM KCl and without or with WDR5. These traces were low-pass filtered at 0.1 kHz. (b) The top panel shows a standard event histogram of τ_on_ in 50 mM KCl with WDR5.τ_on_ (mean ± s.e.m.) was 347 ± 9 ms (number of events: *N* = 801). The bottom panel illustrates a standard event histogram of τ_off_ in 50 mM KCl.τ_off_ (mean ± s.e.m.) was 153 ± 20 ms (*N* = 775). (c) Same as (a) but in 400 mM KCl. Traces were low-pass filtered at 1 kHz. (d) Same as (b) but in 400 mM KCl. The top panel shows an event histogram of unbound durations. τ_on_ (mean ± s.e.m.) was 844 ± 30 ms (number of events: *N* = 446). The bottom panel illustrates a standard event histogram of bound durations. τ_off_ (mean ± s.e.m.) was 33 ± 3 ms (*N* = 461). In (a–d), [WDR5] was 11.4 μM. (e) Semilogarithmic representation of the dependence of the associated rate constant (*k*_on_) on [WDR5]. (f) Dependence of *k*_off_ (1*/τ*_off_) on [KCl]. For (a) and (c), *O*_on_ and *O*_off_ indicate the unbound and bound substates, respectively. All single-channel electrical recordings were replicated in at least *n* = 3 independent experiments. The applied transmembrane potential was −20 mV. All single-channel electrical recordings were conducted at varying [KCl], with a solution containing 20 mM Tris-HCl, 1 mM TCEP, and pH 7.5.

In this study, we decreased the KCl concentration, [KCl], of the solution from 300 to 50 mM while keeping pH 7.5. This drastic change in the [KCl] value substantially increased the Debye–Hückel screening length, λ_D_, from 0.56 nm at 300 mM KCl to 1.4 nm at 50 mM KCl (Table S8). Despite an extensive reduction in the open-state current of MYC_WBM_tFhuA at 50 mM KCl, we could have accurately detected MYC_WBM_–WDR5 binding events (Figure 4a,b; able S9 and Figure S5). A stronger binding affinity of the MYC_WBM_–WDR5 interaction was noted through an increase in the frequency of bound events and an amplification in the duration of bound events. We also examined the WDR5 recognition events at 400 mM KCl (Figure 4c,d). In this case, we observed decreased frequency and duration of bound events, resulting in a substantially weaker interaction.

This trend of the frequency and duration of bound events was replicated for all explored [KCl] values (Figure 4e,f; Figures S6 and S7 and Tables S10–S12). A relatively modest alteration in these results was noted between 200 and 400 mM KCl, a salt concentration range corresponding to a somewhat limited change in the Debye screening length, λ_D_ (Table S8).^67^ In 400 mM KCl, the MYC_WBM_tFhuA sensor detected the weakest MYC_WBM_–WDR5 interaction with a *K*_D_ of 0.29 ± 0.01 mM (Table S13). In 50 mM KCl, we determined a *K*_D_ of 25 ± 5 μM, indicating a 10-fold stronger interaction than that acquired in 400 mM KCl. Therefore, we observe that the MYC_WBM_–WDR5 complex is, to some extent, electrostatically driven (Figures S8 and S9).

Unexpectedly, the change in the binding affinity, within the 50–400 mM KCl range, was less than most electrostatically amplified interactions.^62^ For example, the barnase-barstar complex^62,68,69^ exhibits a 10-fold alteration in the *k*_on_ and a 4-fold change in the *k*_off_ for the same range of electrolyte concentrations.^70^ In contrast, we only observed a 3-fold increase in the *k*_on_ and a similar decline in the *k*_off_ for the MYC_WBM_–WDR5 complex when [KCl] was reduced from 400 to 50 mM KCl. The electrolyte-free value of the *k*_on_ was (2.9 ± 0.1) × 10^5^ M^–1^ s^–1^, as determined from the intercept of the linear fit of the function *k*_on_([KCl]) with the vertical axis (Figure 4e). In a semilogarithmic representation, the *k*_on_ followed a linear relationship with the mean activity coefficient of the electrolyte, *f*_±_^*^ (Figure S8),^71,72^ as expected for electrostatically mediated interactions.^73^

The Bjerum length, *l*_B_ (∼0.71 nm), is given by the following equation
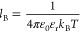
1where ε_0_, ε_r_, *k*_B_, and *T* are the electric permittivity of vacuum, relative electric permittivity with respect to vacuum, Boltzmann’s constant, and absolute temperature, respectively. The Bjerrum length is the separation distance of two oppositely charged monovalent ions at which the electrostatic interaction energy equals the thermal energy. The Debye screening length, λ_D_, provides the range of the electrostatic energy between two ions in an electrolyte solution. If the electrolyte concentration, *I*, increases, its value decreases. For two oppositely charged monovalent ions in water at 25 °C,^74^
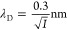
2

Hence, we expect two regimes, one at *l*_B._ < λ_D_, where the electrostatic energy is dominant, and one at *l*_B_ > λ_D_, where the thermal energy is dominant.^62^ Using eq 2, for KCl solutions at room temperature, the boundary between the two regimes corresponds to 178.5 mM KCl (Table S8). Surprisingly, the two regimes are not obviously apparent in Figure 4e,f. One possible interpretation of this finding is the significant decline in the *k*_on_ change over the range of [KCl] between 50 and 400 mM with respect to electrostatically assisted associations.^73^ As discussed below, the short-range hydrophobic contacts may be responsible for the reduced effect of the [KCl] alterations on the *k*_on_. Overall, this interaction is not solely mediated by electrostatic forces. Hence, the [KCl]-dependent changes in the *k*_on_ are dampened.

### Composite Effect of the Hydrophobic and Electrostatic Contacts

Prior studies by Tansey and co-workers^6^ highlighted the importance of hydrophobic contacts that stabilize the MYC_WBM_–WDR5 interaction (Table S1). The WBM site of WDR5 encompasses a hydrophobic core flanked by a positive charge, which interacts via ion-pair contacts with the negative charge of the MYC_WBM_ core (EEEIDVV). This electrostatic contribution is supplemented by two stabilizing hydrophobic pockets of WBM exposed to the isoleucine and valine side chains of the MYC_WBM_ core (Figure 1b). The WDR5 residues Y228, L240, and L249 in one pocket accommodate I7 of MYC_WBM_. In the other pocket, the WDR5 residues F266 and V268 form hydrophobic contacts with V9 and V10 of MYC_WBM_. These hydrophobic forces are likely responsible for the relatively reduced changes in the *k*_on_ with respect to systematic alterations in the ionic strength.

Hence, the MYC_WBM_–WDR5 complex is an interaction that exhibits an interplay between enhanced hydrophobic contacts and reduced electrostatic interactions as the KCl concentration is increased. Indeed, the hydrophobic forces at the MYC_WBM_–WDR5 interface are less exposed at increased electrolyte concentrations due to an enhancement in the surface free energy of nonpolar groups via their desolvation.^75^ The basal value of the *k*_on_ at infinite [KCl] in the absence of electrostatic forces was roughly lower than 10^5^ M^–1^ s^–1^ (Figure 4e). Suppose the basal value for electrostatically enhanced peptide–protein interactions is equal to or greater than ∼8 × 10^5^ M^–1^ s^–1^.^76^ In that case, the short-range hydrophobic contacts^62^ of the MYC_WBM_–WDR5 interaction contribute to an increase in the activation free energy by at least *RT* × ln(8) ≅ 2.1 × *RT,* where *R* and *T* are the general gas constant and absolute temperature. Next, we qualitatively validated the weak nature of the MYC_WBM_–WDR5 interaction via ND-BLI. However, quantitative kinetic values could not be extracted due to BLI’s limited time-bandwidth (Figure S10). Again, this finding highlights the power and sensitivity of our engineered nanopore sensor for probing the weak MYC_WBM_–WDR5 interaction under various experimental circumstances.

### Voltage Dependence of the MYC_WBM_–WDR5 Interaction

**Figure 5:**
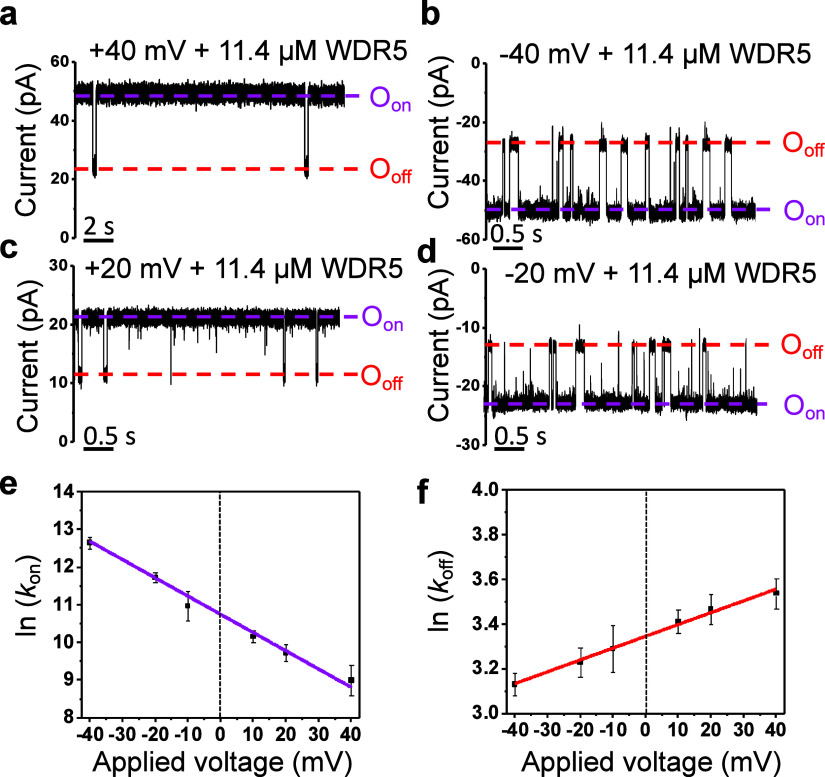
Voltage dependence of the MYC_WBM_–WDR5 interaction. (a) Single-channel recording with an applied voltage of +40 mV. (b) Same as (a) but with an applied voltage of −40 mV. (c) Same as (a) but with an applied voltage potential of +20 mV. (d) Same as (a) but with an applied voltage of −20 mV. In (a–d), *O*_on_ and *O*_off_ indicate the unbound and bound substates, respectively. All single-channel electrical signatures were replicated in *n* = 4 independent experiments and in the presence of 11.4 μM WDR5. Single-channel electrical traces were low-pass filtered at 1 kHz using an 8-pole Bessel filter. All recordings were conducted in 300 mM KCl, 20 mM Tris-HCl, 1 mM TCEP, and pH 7.5. (e) LOinear plot showing the dependence of ln(*k*_on_) on the applied voltage. (f) Linear plot presenting the dependence of ln(*k*_off_) on the applied voltage. Data points in (e) and (f) indicate mean ± s.d. obtained from *n* = 4 distinct experiments.

Next, we examined the voltage dependence of the MYC_WBM_–WDR5 complex formation to understand better this physical factor’s influence on the interaction strength. These experiments were conducted in 300 mM KCl, 20 mM Tris-HCl, 1 mM TCEP, and pH 7.5, using 11.4 μM WDR5 added to the *cis* side. These experimental conditions yielded the best signal-to-noise ratio and a relatively high number of bound events, warranting the acquisition of statistically significant mean parameters. A substantial increase in the frequency of bound events was observed when the applied transmembrane potential was varied from +40 to −40 mV (Figure 5a–d; Tables S14–S15 and Figures S11 and S12).

This sensitivity to the polarity of the applied transmembrane potential was likely due to the overall positive charge of WDR5. WDR5 was attracted closer to the pore at a negative transmembrane potential, facilitating an increased *k*_on._ In addition, we observed a linear decrease in ln (*k*_on_) by amplifying the applied transmembrane potential (Figure 5e). The *y*-intercept of ln(*k*_on_) versus the applied potential provides the association rate constant of the MYC_WBM_–WDR5 interaction at 0 mV, *k*_on_(0 mV), which was (0.48 ± 0.01) × 10^5^ M^–1^ s^–1^ (Table S15). A subtle increase in the *k*_off_ was observed as the applied transmembrane potential was elevated (Figure 5f). The changes in the activation free energy, Δ*G*_on_, for the WDR5-unbound events with respect to a zero applied transmembrane potential, ΔΔ*G*_on,_ were −1.1 ± 0.1 and 1.0 ± 0.1 kcal/mol at −40 and +40 mV, respectively (Table S16). Using this voltage-dependent kinetic data, we determined the apparent net charge, *z*, of WDR5 as 1.3 ± 0.2 (Table S17).

### Comparisons between MYC_WBM_–WDR5 and MLL4_Win_–WDR5 Interactions

**Figure 6:**
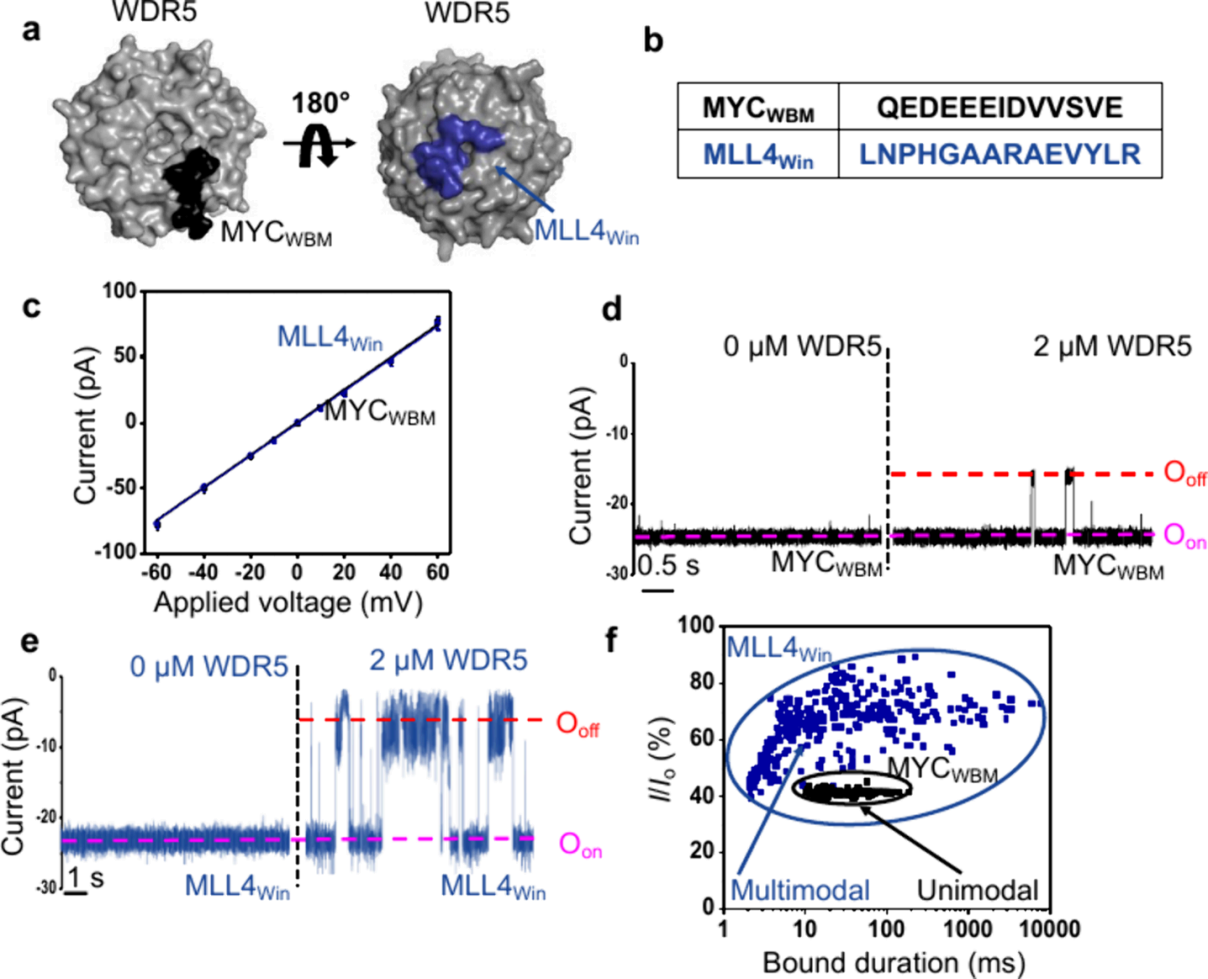
MYC_WBM_–WDR5 and MLL4_Win_–WDR5 interactions detected with nanopore sensors. (a) MYC_WBM_–WDR5 (PDB: 4Y7R)^6^ and MLL4_Win_–WDR5 (PDB: 4ERZ)^77^ interactions. WBM and Win sites are located on opposite sides of the donut-shaped WDR5. (b) Sequences of MYC_WBM_ and MLL4_Win_. (c) These plots indicate the open-state current as a function of the applied voltage for MYC_WBM_tFhuA (black squares) and MLL4_Win_tFhuA (blue circles). Data points represent mean ± s.d. obtained from *n* = 4 independent experiments. (d) Recording of MYC_WBM_tFhuA without and with 2 μM WDR5. The applied transmembrane potential was −20 mV. Single-channel electrical traces were low-pass filtered at 1 kHz. (e) Same as (d) but with MLL4_Win_tFhuA. (f) *I*/*I*_0_ as a function of WDR5-bound duration probed by MYC_WBM_tFhuA and MLL4_Win_tFhuA in the presence of 2 μM WDR5. *I*_0_ and *I* denote the open-state currents and the current amplitudes of WDR5-produced blockades, respectively. The WDR5-bound events for MYC_WBM_tFhuA and MLL4_Win_tFhuA were marked in black (unimodal protein recognition) and blue (multimodal protein recognition), respectively.

In this work, we evaluate the WBM-mediated interaction of WDR5 with the MYC_WBM_ motif. Yet, WDR5 features another binding interface in the form of an acidic central cavity, called the WDR5 interaction (Win) site (Figure 6a).^66,78^ The two binding sites of WDR5 provide a unique opportunity to compare distinctive interactions of this nuclear hub. Hence, we were able to compare the data using the MYC_WBM_tFhuA nanopore sensor to a previously developed sensor that contained a consensus peptide ligand of mixed lineage leukemia 4 (MLL4)^79−81^ methyltransferase against the Win site (MLL4_Win_)^77,82^ (Figure 6a,b). We named this nanopore sensor as MLL4_Win_tFhuA.^83^ As expected, both sensors showed a closely similar unitary current (Figure 6c; Tables S2 and S18). MYC_WBM_tFhuA and MLL4_Win_tFhuA exhibited quiet electrical traces without WDR5 but drastically different single-channel responses in the presence of WDR5 (Figure 6d,e). Specifically, the current blockades due to the MLL4_Win_-WDR5 interaction were far less uniform than those observed through the MYC_WBM_–WDR5 interaction (Figures S13 and S14). Three distinct subpopulations of WDR5 capture events were observed with MLL4_Win_tFhuA (Table S19). They were noted through short-, medium-, and long-lived current blockades, as denoted by subscripts “1″, “2″, and “3″, respectively.

The largest association rate constant, *k*_on-1,_ corresponding to short-lived events, was (1.4 ± 0.1) × 10^5^ M^–1^ s^–1^. Notably, the dissociation rate constants, *k*_off-i_ (i = 1, 2, 3), resulted from WDR5-bound times that span at least 3 orders of magnitude between low-millisecond durations and very long-lived captures measuring tens of seconds. They were also distinct from the *k*_off_ determined for the MYC_WBM_ – WDR5 interaction. These vastly diverse Win-mediated WDR5 captures illustrated a multimodal protein recognition of WDR5 using MLL4_Win_tFhuA (Table S20). In contrast, MYC_WBM_tFhuA revealed a unimodal protein recognition of WDR5 via the WBM site. These findings reinforce the high sensitivity of this biosensing approach in protein analytics, illuminating kinetic complexities of the protein hub recognition that are otherwise hidden in measurements by existing technologies in an ensemble.

The two sensors also exhibited a different mean normalized current amplitude (*I*/*I*_0_) of WDR5-produced current blockades (Figure 6f and Table S21). *I* and *I*_0_ are the current amplitudes of WDR5-induced blockades and the open state, respectively. Like the broad range of WDR5 captures detected for the MLL4_Win_-WDR5 interaction, *I*/*I*_0_ spanned between 40%–95%. On the contrary, the *I*/*I*_0_ for the MYC_WBM_–WDR5 interaction converged to a peak at 41 ± 3%. In addition, prior voltage-dependent experiments conducted with the MLL4_Win_tFhuA sensor^83^ determined a smaller relative charge of WDR5 than that determined in this study using MYC_WBM_tFhuA, which is in accordance with more positively charged residues surrounding the WBM site than the Win site (Table S22 and Figure S15). Moreover, we also evaluated the MLL4_Win_-WDR5 interaction via ND-BLI and qualitatively confirmed that the MLL4_Win_-WDR5 interaction was stronger than the MYC_WBM_–WDR5 interaction (Figure S16 and Table S23).

### Multitasking Roles of WDR5 and MYC

WDR5 is notorious for its implications in regulatory mechanisms of gene expression via large multisubunit epigenetic complexes. For example, WDR5 interacts with a high affinity with each of the six human SET1/MLL histone methyltransferases (MLL1–4 and SETd1A-B) for H3K4 methylation using the Win site,^66,77,78,82^ which is located within a central acidic cavity (Figure S15). In addition, it has been shown that the Win site mediates transient PPIs with dozens of proteins, including those involved in key signaling pathways, such as phosphatidylinositol 3-kinase (PI3K) and 3-phosphoinositide-dependent protein kinase 1 (PDPK1).^84^ Interestingly, a high-density distribution of missense somatic cancer mutations of WDR5 occurs within and around the Win site,^85^ some of which significantly alter the strength of the MLL-WDR5 interactions.^78,83,86,87^

On the opposite side of the Win site, WDR5 includes the WBM binding site. This hydrophobic cleft facilitates a weak-affinity interaction with the retinoblastoma-binding protein 5 (RbBP5),^15^ a subunit of the SET1/MLL complexes. In addition, WBM serves as a binding site beyond the contexts of large epigenetic complexes. This includes interactions that promote the recruitment of MYC to chromatin.^5−7^ Furthermore, MYC is involved in numerous PPIs, which are critical to regulatory mechanisms of gene expression. Specifically, the MYC interactome encompasses 336 binding proteins implicated in direct physical associations with at least six evolutionarily conserved MYC homology boxes.^88^ Through a complex interactome, MYC is a focal element that coordinates many cellular signals that result from a wide transcriptional spectrum.^89,90^ For example, MYC regulates essential processes of gene transcription, such as epigenetic modifications, as well as promoter binding, initiation, elongation, and posttranscriptional events. Using different approaches, we^4^ and others^6^ found that MYC_WBM_ weakly interacts with WDR5. One immediate question is whether the full-length MYC also exhibits a weak affinity against the full-length WDR5. In an independently executed fluorescence resonance energy transfer (FRET) study in living cells,^4^ a weak MYC-WDR5 interaction was confirmed through a low FRET efficiency of the complex formation. MYC recruitment to chromatin is facilitated by its coordinated interactions with an obligate MAX binding partner, a specific E-box DNA sequence, and a prebound WDR5 molecule.^8^ The association kinetics of the MYC-WDR5 complex is limited by the diffusion of the MYC:MAX heterodimer to chromatin and the immobilized WDR5 protein. In addition, the MYC-WDR5 interaction may also be affected by the crowding subnuclear environment around chromatin and other physical restraints,^91^ such as WDR5′s anchoring system to chromatin via the Win site.^8^ Yet, we do not expect significant alterations in the kinetic and affinity parameters in the presence of chromatin with respect to the data provided in this study.

### Advantages of This Single-Polypeptide Chain Protein Nanopore in Biosensing Technology

In this study, we created and validated an MYC_WBM_-containing biological nanopore for probing WDR5 via a binding site involved in the molecular mechanisms of tumorigenesis. This single-polypeptide chain sensing element samples a highly specific but weak interaction of a protein hub in a real-time and label-free setting. The genetically encoded nature of this engineered nanopore enables further advances of other sensors comprising combinatorial libraries of tethered peptide ligands. This way, such an experimental strategy will maintain their architecture and increased sensitivity while substantially expanding their applications to multiple hot spots of the same protein hub.

With further developments, such nanopore sensors can be created for screening small-molecule inhibitors aimed at targeting WDR5-mediated interactions using the Win and WBM sites, as previously reported against the Pim kinase.^92^ For example, MYC is a challenging therapeutic target^93^ due to its extended intrinsically disordered regions and complex regulatory processes. In the past several years, experimental evidence has proven that disrupting the MYC-WDR5 interaction via small-molecule inhibitors is a potentially effective mechanism for inhibiting tumorigenesis.^7,8^ Hence, these sensing elements may have an influential impact on drug development pipelines and cancer nanomedicine. In addition, they can be formulated as single-molecule enzyme detectors. There is no fundamental limitation in substituting the MYC_WBM_ peptide with any pseudosubstrate peptide against a clinically relevant serine-threonine kinase (STK).^45^ This way, the dependence of the STK activity and interactions on cofactors, cosubstrates, and inhibitors for the phosphate transfer can be directly determined without requiring any exogenous fluorophore or chemically attached reporters. However, kinase cancer therapeutics utilizing inhibitors are usually challenged by acquired resistance, and concurrent therapies expectedly result in amplified drug toxicity. Therefore, this method may generate a route to identify a minimal clinical dose of an inhibitor. Finally, the throughput of this approach may be scaled up by pairing these high-resolution electrical measurements with parallel recording technologies.

Future advancements may also take this proof of concept to a different level in protein analytics. Because of their monomeric structure, engineering these sensors with a targeted peptide ligand outside the pore lumen can be accomplished without tedious purification protocols, such as those in the case of multimeric nanopore assemblies. Further, we show that the hub recognition events are discriminated against without the necessity of utilizing complex data analysis algorithms. Other benefits of this sensor design include the following: (i) the characterization of protein interactions can be pursued beyond the fundamental limit of sensing inside the nanopore, (ii) the opportunity to conduct protein binding experiments at a very low salt concentration due to superior signal performance, (iii) the ability to resolve complex kinetics and multimodal protein recognition, which are otherwise undetectable by existing methods in an ensemble, and (iv) the amenability to ultrasensitive determinations of high *k*_off_ values that other real-time kinetic techniques cannot quantitatively assess.

## Conclusions

In summary, we determined the detailed kinetics of the reversible MYC_WBM_–WDR5 interaction at single-molecule resolution. In this study, we provided an additional evaluation of this interacting pair to show that it is, to some extent, electrostatically enhanced, yet its hydrophobic contacts limit this influence. Also, we quantitatively and qualitatively compared the nature and strength of the MYC_WBM_–WDR5 and MLL4_Win_–WDR5 interactions, which are mediated by different binding sites of the same hub. The two interaction interfaces produced vastly distinctive electrical and protein recognition signatures. These outcomes, which were inferred utilizing the same nanopore architecture, highlight the uniqueness of each binding site’s kinetic fingerprint and the undeniable sensitivity of our biosensing approach. Finally, this work can serve as a roadmap for future evaluations of weak interactions between transcription factors and their corresponding protein cofactors.

## Experimental Section

### Nanopore Sensor Equipped with the MYC_WBM_ Peptide Ligand

A plasmid containing the *omyctfhua* gene was derived from the *omll4tfhua* gene. The *omll4tfhua* gene was purchased from GenScript (Piscataway, NJ).^83^ At the N terminus, each gene also included a sequence encoding a peptide ligand for interacting with WDR5 through one of its binding sites and a 13-residue peptide adaptor (O, MGDRGPEFELGTM).^94^ The *mll4* gene encoded a 14-residue Mixed Lineage Leukemia 4 (MLL4) WDR6 interaction (Win) motif peptide ligand (MLL4_Win_, LNPHGAARAEVYLR).^77,82^ A site-directed mutagenesis kit (New England Biolab, Ipswich, MA) was utilized to substitute the *mll4* gene with the *myc* gene. MLL4_Win_ was replaced with a 13-residue MYC WDR5 binding motif (WBM) peptide ligand (MYC_WBM_, QEDEEEIDVVSVE).^6^ In each case, a 6-residue Gly/Ser-rich peptide tether was used to fuse the peptide ligand to the 455-residue extensive truncation of *ferric hydroxamate uptake component* A of *Escherichia coli* (tFhuA). Finally, we deleted the adaptor sequence from the original gene sequence to form *myctfhua*. The pPR-IBA1 vector was used for all templates.^95,96^

These plasmids were transformed into *E. coli* BL21(DE3) cells. After the successful transformation, cells were grown in a Luria–Bertani medium at 37 °C until OD_600_ was ∼0.4. Cells were induced by adding 1 mM isopropyl β-d-1-thiogalactopyranoside (IPTG). They were grown further for ∼5 h at 37 °C. Cells were centrifuged at 3,700 × *g* and 4 °C for 30 min. This process was followed by their resuspension into a buffer containing 300 mM KCl, 20 mM Tris-HCl, 5 mM ethylenediaminetetraacetic acid (EDTA), and pH 8. A Model 110 L microfluidizer (Microfluidics, Newton, MA) was used to lyse cells. The lysate was centrifuged at 108,500 × *g* for 30 min at 4 °C. The supernatant was removed, and the targeted nanopore sample remained in the pellet. The pellets underwent a series of 1.5% Triton X-100, 1 mM EDTA washes to remove impurities. The final precipitate was solubilized in 8 M urea for a further purification protocol.

### Purification and Refolding of the MYC_WBM_tFhuA Nanopore Sensors

The purification of nanopore proteins began with a run on an anion-exchange column (Q12-Sepharose; Bio-Rad, Hercules, CA). This chromatography utilized a linear gradient of 0–1 M KCl, 20 mM Tris-HCl, pH 8. To remove protein precipitates, the peak fractions were extracted and centrifuged at 3,700 × *g* for 10 min at 4 °C. These samples were passed through a size-exclusion column (SEC, HiLoad16/600 Superdex-75; GE Healthcare Life Sciences, Pittsburgh, PA) for a final purification step. The buffer was 200 mM KCl, 8 M urea, 20 mM Tris-HCl, and pH 8. The purified protein came out in fractions at the correlated target size. Protein aggregates were removed through centrifugation at 3,700 × *g* for 10 min at 4 °C. The protein purity was analyzed using SDS-PAGE analyses, and then pure samples were used further for detergent-mediated refolding.

The purified sample was brought to a final concentration between 20 and 24 μM. The denatured samples were incubated in *n*-dodecyl-β-D-maltopyranoside (DDM; Anatrace, Maumee, OH) to a final concentration of 1% (w/v). After 5 min of mixing at room temperature, the solubilized protein was added to a dialysis bag with a 14 kDa-molecular weight cutoff. The sample was dialyzed at 4 °C against 200 mM KCl, 20 mM Tris-HCl, and pH 7.5. The dialysis solution was refreshed once every 24 h for 72 h. To remove protein aggregates, the refolded protein sample was centrifuged at 3,700 × *g* for 5 min. The refolded protein was in the supernatant. Protein quantification was determined using the molar absorptivity at a wavelength of 280 nm, and the samples were used for single-channel electrical recordings.

### Expression and Purification of WDR5

An N-terminal truncation of WDR5 (WDR5^23–334^ or WDR5_ΔN_) was expressed using Rosetta II pLysS competent cells (Novagen via Millipore Sigma, Burlington, MA). For the sake of simplicity, we will use this variant named WDR5 throughout this article. The WDR5-encoding plasmid was a gift from Michael S. Cosgrove. After the successful transformation, cells were grown in Luria–Bertani medium at 37 °C until OD_600_ was ∼0.75. Then, they were chilled at 4 °C until they reached an OD_600_ value of ∼1.0. The cells were induced using 1 mM IPTG. Then, cells were grown for 18–20 h at 16 °C. Growth of cells was followed by harvesting through centrifugation at 4,000 × *g*. This step was conducted at 4 °C for 30 min. The cellular pellet was resuspended in the lysis buffer, which contained 300 mM KCl, 50 mM Tris, 3 mM dithiothreitol (DTT), 30 mM imidazole, 0.1 mM phenylmethylsulfonyl fluoride (PMSF), and pH7.4. This buffer also included one EDTA-free protease inhibitor cOmplete cocktail tablet (Sigma-Aldrich, St. Louis, MO). A microfluidizer (Model M110L; Microfluidics, Newton, MA) was used to lyse the cells. The lysate was centrifuged at 108,500 × *g* at 4 °C for 35 min to clear cellular debris. The WDR5-containing supernatant was loaded into a 5 mL-volume metal-affinity column (Bio-Scale Mini Profinity IMAC cartridge; Bio-Rad, Hercules, CA).

After the initial pass over the metal-affinity column, an SDS-PAGE gel was run to determine the collected protein fractions. To remove the hexahistidine tag, the pure protein samples were exposed to Tobacco Etch Virus (TEV) protease (New England Biolabs, Ipswich, MA). After the TEV digestion, the protein sample was dialyzed to reduce imidazole concentration to 30 mM. Then, it was passed over the immobilized metal-affinity column for a second run. The purified WDR5 samples were concentrated using a spin concentrator (10 kDa-molecular weight cutoff; Millipore Sigma, St. Louis, MO). The final sample was dissolved in a buffer containing 300 mM KCl, 50 mM Tris-HCl, 1 mM tris(2-carboxyethyl)phosphine (TCEP), and pH 7.5.

### Electrical Recordings Using Planar Lipid Membranes

Single-channel electrical recordings exploring functionally reconstituted nanopore sensors into lipid membranes were conducted, as previously reported.^97^ A 90 μm-diameter aperture was created in a 25 μm-thick Teflon film (Goodfellow Corporation, Malvern, PA), which supported the planar lipid bilayers. The Teflon aperture was pretreated with hexadecane (Sigma-Aldrich, St. Louis, MO), dissolved in pentane (Fisher HPLC grade, Fair Lawn, NJ). This pretreatment facilitated the formation of a lipid bilayer across the aperture in the Teflon film. The planar lipid bilayer was made of 1,2-diphytanoyl-*sn*-glycero-phosphatidylcholine (Avanti Polar Lipids, Alabaster, AL). Unless otherwise indicated, experiments were conducted in 300 mM KCl, 20 mM Tris-HCl, 1 mM TCEP, and pH 7.5 at room temperature (23 ± 1 °C). Protein samples were added to the grounded side of the electrical chamber, the *cis* side, at a final concentration of ∼1 ng/μL. WDR5 was also titrated into the *cis* side at indicated concentrations. A patch-clamp amplifier (Model Axopatch 200B, Axon Instruments, Foster City, CA) was used to conduct single-channel electrical recordings. The analog signal was low-pass filtered (8-pole Bessel filter, Model 900; Frequency Devices, Ottawa, IL), then digitized utilizing a low-noise acquisition system (Model Digidata 1440 A; Axon Instruments). The digitized signal was sampled at 50 kHz. For the data analysis, electrical traces were filtered at 1 kHz.

### Single-Channel Statistical Analysis

Single-channel data was acquired and analyzed via pClamp 10.7 (Axon Instruments) and ClampFit 10.7 (Axon Instruments), respectively. All investigations resulted from 10 min recordings unless otherwise stated. Multiple fitting models were tested for WDR5-bound and unbound durations. A kinetic rate matrix was used to produce each model’s probability distribution function (PDF). The maximum likelihood method (MLM)^60^ and logarithm likelihood ratio (LLR)^61^ tests were employed to determine the number of statistically significant event subpopulations best suited to the data. At a confidence number of 0.95, the best model for the WDR5-unbound durations was a single-exponential fit for MYC_WBM_–WDR5 and MLL4_Win_-WDR5 interactions. The best model for the WDR5-bound durations was a single-exponential fit for the MYC_WBM_–WDR5 interaction. However, the best model for the WDR5-bound durations was a three-exponential fit for the MLL4_Win_-WDR5 interaction.

### Nanopore–Nanodisc Complexation for the Bulk-Phase Optical Sensing

Before reconstitution, the nanodiscs (NDs) were fabricated from biotinylated membrane scaffold proteins (MSP).^94,98^ The reconstitution of MYC_WBM_tFhuA occurred in one step. The detergent-solubilized MYCWBMtFhuA was combined with biotinylated MSP at a ratio of 1:2, respectively. The detergent, *n*-dodecyl-β-d-maltopyranoside (DDM; Anatrace), was maintained at a constant concentration of 1% (v/v). Also, 1,2-diphytanoyl-*sn*-glycero-phosphatidylcholine lipids (Avanti Polar Lipids) were added to the mix at a 1:2:4 MYC_WBM_tFhuA:MSP:lipid ratio. This mixture was left to rotate at 4 °C for 1 h. Then, the detergent was removed by adding 0.4 g/mL of the activated biobeads. The detergent extraction was conducted for 2 h of constant rotation at 4 °C. Then, the activated biobeads were removed after they were separated from the supernatant during centrifugation at 5,000 × *g* at 4 °C for 5 min. The supernatant was run on a size-exclusion column to collect the elution peaks with the nanodisc-reconstituted MYC_WBM_tFhuA.

### Biolayer Interferometry

All biolayer interferometry (BLI) experiments were conducted utilizing an Octet Red384 platform (FortéBio, Fremont, CA).^91,99^ The MYC_WBM_tFhuA nanosensor was reconstituted into a biotinylated lipid nanodisc (ND). Streptavidin (SA) sensors were washed with 20 mM Tris-HCl, 300 mM KCl, 1 mM TCEP, 1 mg/mL bovine serum albumin (BSA), and pH 7.5 for 30 min. Fifteen nM biotinylated MYC_WBM_tFhuA-containing NDs were loaded onto the SA sensors for 15 min. The unbound NDs were removed by dipping the sensors into 20 mM Tris-HCl, 300 mM KCl, 1 mM TCEP, 1 mg/mL BSA, and pH 7.5 for 5 min. A serial dilution of WDR5 between 2–18 μM was added to inspect the WDR5-MYC_WBM_tFhuA association process. Then, the dissociation process was evaluated by placing the BLI sensors into a WDR5-free buffer. For all experiments, ND-free BLI sensors were also independently run as controls. These controls were employed to subtract the baseline and drift in the sensorgrams to determine the binding curves. These BLI experiments were conducted at 24 °C.

### Steady-State Fluorescence Polarization (FP) Anisotropy

As previously reported, FP studies were carried out using a 96-well plate reader (SpectraMax i3; Molecular Devices, San Jose, CA).^64,65^ All runs were conducted in a buffer containing 150 mM NaCl, 20 mM Tris-HCl, 1 mM TCEP, and 0.005% tween 20, pH 7.5. Serially diluted concentrations of WDR5 were loaded onto a 96-well plate as the analyte, and 50 nM fluorescein isothiocyanate (FITC)-labeled peptides were added as ligands. The anisotropy values were measured after a 1 h incubation at room temperature. The dose–response curve was fitted with a four-parameter logistic curve to extract the interactions’ equilibrium dissociation constant, *K*_D_.

### Molecular Graphics

All figures showing molecular representations were prepared using PyMOL V2.4.0 (Schrödinger, LLC, New York, NY). Entries 4Y7R^6^ and 4ERZ^77^ from Protein Data Bank were used for visualizations and molecular graphics of the MYC_WBM_–WDR5 and MLL4_Win_-WDR5 complexes, respectively.

## Supporting Information Available

List of hydrogen bonds, hydrophobic contacts, and ionic bonds at the MYC_WBM_–WDR5 interface, kinetic rate constants, equilibrium dissociation constant, and BLI sensorgrams for the MYC_WBM_–WDR5 interaction, steady-state FP curves of the MYC_WBM_–WDR5 interaction, single-channel electrical traces acquired in the presence and absence of WDR5 using MYC_WBM_tFhuA under various salt concentrations, corresponding event duration histograms, association and dissociation rate constants, equilibrium dissociation constant, and BLI sensorgrams for the MYC_WBM_–WDR5 interaction at various [KCl] values, association and dissociation rate constants for the MYC_WBM_–WDR5 interaction at various applied voltages, reduction in activation free energies of WDR5-unbound events at different transmembrane potentials, relative charge of WDR5 determined by voltage dependence experiments, single-channel electrical traces of MLL4_Win_tFhuA when WDR5 was added to the *cis* compartment and corresponding event duration histograms, kinetic rate constants determined for the WDR5 detection with MLL4_Win_tFhuA and MYC_WBM_tFhuA, mean normalized current amplitude for MLL4_Win_tFhuA and MYC_WBM_tFhuA nanopores, and BLI sensorgrams of the MYC_WBM_ and MLL4_Win_ interactions with WDR5 (PDF)

## References

[ref1] Lambert S. A.; Jolma A.; Campitelli L. F.; Das P. K.; Yin Y.; Albu M.; Chen X.; Taipale J.; Hughes T. R.; Weirauch M. T. The Human Transcription Factors. Cell 2018, 172 (4), 650–665. 10.1016/j.cell.2018.01.029. 29425488 PMC12908702

[ref2] Suter D. M. Transcription Factors and DNA Play Hide and Seek. Trends Cell. Biol. 2020, 30 (6), 491–500. 10.1016/j.tcb.2020.03.003. 32413318

[ref3] Göös H.; Kinnunen M.; Salokas K.; Tan Z.; Liu X.; Yadav L.; Zhang Q.; Wei G. H.; Varjosalo M. Human transcription factor protein interaction networks. Nat. Commun. 2022, 13 (1), 766 10.1038/s41467-022-28341-5. 35140242 PMC8828895

[ref4] Ahmad M.; Imran A.; Movileanu L. Overlapping characteristics of weak interactions of two transcriptional regulators with WDR5. Int. J. Biol. Macromol. 2024, 258 (Pt 2), 128969 10.1016/j.ijbiomac.2023.128969. 38158065 PMC10922662

[ref5] Thomas L. R.; Foshage A. M.; Weissmiller A. M.; Tansey W. P. The MYC-WDR5 Nexus and Cancer. Cancer Res. 2015, 75 (19), 4012–4015. 10.1158/0008-5472.CAN-15-1216. 26383167 PMC4592407

[ref6] Thomas L. R.; Wang Q.; Grieb B. C.; Phan J.; Foshage A. M.; Sun Q.; Olejniczak E. T.; Clark T.; Dey S.; Lorey S.; Alicie B.; Howard G. C.; Cawthon B.; Ess K. C.; Eischen C. M.; Zhao Z.; Fesik S. W.; Tansey W. P. Interaction with WDR5 promotes target gene recognition and tumorigenesis by MYC. Mol. Cell. Biochem. 2015, 58 (3), 440–52. 10.1016/j.molcel.2015.02.028. PMC4427524 25818646

[ref7] Thomas L. R.; Adams C. M.; Wang J.; Weissmiller A. M.; Creighton J.; Lorey S. L.; Liu Q.; Fesik S. W.; Eischen C. M.; Tansey W. P. Interaction of the oncoprotein transcription factor MYC with its chromatin cofactor WDR5 is essential for tumor maintenance. Proc. Natl. Acad. Sci. U S A 2019, 116 (50), 25260–25268. 10.1073/pnas.1910391116. 31767764 PMC6911241

[ref8] Thomas L. R.; Adams C. M.; Fesik S. W.; Eischen C. M.; Tansey W. P. Targeting MYC through WDR5. Mol. Cell. Oncol. 2020, 7 (2), 1709388 10.1080/23723556.2019.1709388. 32158922 PMC7051159

[ref9] Ahmadi S. E.; Rahimi S.; Zarandi B.; Chegeni R.; Safa M. MYC: a multipurpose oncogene with prognostic and therapeutic implications in blood malignancies. J. Hematol. Oncol. 2021, 14 (1), 121 10.1186/s13045-021-01111-4. 34372899 PMC8351444

[ref10] Madden S. K.; de Araujo A. D.; Gerhardt M.; Fairlie D. P.; Mason J. M. Taking the Myc out of cancer: toward therapeutic strategies to directly inhibit c-Myc. Mol. Cancer. 2021, 20 (1), 3 10.1186/s12943-020-01291-6. 33397405 PMC7780693

[ref11] Dhanasekaran R.; Deutzmann A.; Mahauad-Fernandez W. D.; Hansen A. S.; Gouw A. M.; Felsher D. W. The MYC oncogene - the grand orchestrator of cancer growth and immune evasion. Nat. Rev. Clin. Oncol. 2022, 19 (1), 23–36. 10.1038/s41571-021-00549-2. 34508258 PMC9083341

[ref12] Llombart V.; Mansour M. R. Therapeutic targeting of ″undruggable″ MYC. EBioMedicine 2022, 75, 103756 10.1016/j.ebiom.2021.103756. 34942444 PMC8713111

[ref13] Das S. K.; Lewis B. A.; Levens D. MYC: a complex problem. Trends Cell. Biol. 2023, 33 (3), 235–246. 10.1016/j.tcb.2022.07.006. 35963793 PMC9911561

[ref14] Ullius A.; Luscher-Firzlaff J.; Costa I. G.; Walsemann G.; Forst A. H.; Gusmao E. G.; Kapelle K.; Kleine H.; Kremmer E.; Vervoorts J.; Luscher B. The interaction of MYC with the trithorax protein ASH2L promotes gene transcription by regulating H3K27 modification. Nucleic Acids Res. 2014, 42 (11), 6901–20. 10.1093/nar/gku312. 24782528 PMC4066752

[ref15] Odho Z.; Southall S. M.; Wilson J. R. Characterization of a novel WDR5-binding site that recruits RbBP5 through a conserved motif to enhance methylation of histone H3 lysine 4 by mixed lineage leukemia protein-1. J. Biol. Chem. 2010, 285 (43), 32967–32976. 10.1074/jbc.M110.159921. 20716525 PMC2963364

[ref16] Guarnaccia A. D.; Tansey W. P. Moonlighting with WDR5: A Cellular Multitasker. J. Clin. Med. 2018, 7 (2), 21 10.3390/jcm7020021. 29385767 PMC5852437

[ref17] Nogal B.; Bowman C. A.; Ward A. B. Time-course, negative-stain electron microscopy-based analysis for investigating protein-protein interactions at the single-molecule level. J. Biol. Chem. 2017, 292 (47), 19400–19410. 10.1074/jbc.M117.808352. 28972148 PMC5702678

[ref18] De Keersmaecker H.; Camacho R.; Rantasa D. M.; Fron E.; Uji-i H.; Mizuno H.; Rocha S. Mapping Transient Protein Interactions at the Nanoscale in Living Mammalian Cells. ACS Nano 2018, 12 (10), 9842–9854. 10.1021/acsnano.8b01227. 30192513

[ref19] Lee H. W.; Choi B.; Kang H. N.; Kim H.; Min A.; Cha M.; Ryu J. Y.; Park S.; Sohn J.; Shin K.; Yun M. R.; Han J. Y.; Shon M. J.; Jeong C.; Chung J.; Lee S. H.; Im S. A.; Cho B. C.; Yoon T. Y. Profiling of protein-protein interactions via single-molecule techniques predicts the dependence of cancers on growth-factor receptors. Nat. Biomed. Eng. 2018, 2 (4), 239–253. 10.1038/s41551-018-0212-3. 30936439

[ref20] Sackmann B.; Neher E. Single-Channel Recording; 2nd ed.; Kluwer Academic/Plenum Publishers: New York, 1995.

[ref21] Restrepo-Perez L.; Joo C.; Dekker C. Paving the way to single-molecule protein sequencing. Nat. Nanotechnol. 2018, 13 (9), 786–796. 10.1038/s41565-018-0236-6. 30190617

[ref22] Cressiot B.; Bacri L.; Pelta J. The Promise of Nanopore Technology: Advances in the Discrimination of Protein Sequences and Chemical Modifications. Small Methods 2020, 4 (11), 2000090 10.1002/smtd.202000090.

[ref23] Tripathi P.; Firouzbakht A.; Gruebele M.; Wanunu M. Threading single proteins through pores to compare their energy landscapes. Proc. Natl. Acad. Sci. U. S. A. 2022, 119 (39), e2202779119 10.1073/pnas.2202779119. 36122213 PMC9522335

[ref24] Tanimoto I. M. F.; Cressiot B.; Greive S. J.; Le Pioufle B.; Bacri L.; Pelta J. Focus on using nanopore technology for societal health, environmental, and energy challenges. Nano Res. 2022, 15 (11), 9906–9920. 10.1007/s12274-022-4379-2. 35610982 PMC9120803

[ref25] Ying Y. L.; Hu Z. L.; Zhang S.; Qing Y.; Fragasso A.; Maglia G.; Meller A.; Bayley H.; Dekker C.; Long Y. T. Nanopore-based technologies beyond DNA sequencing. Nat. Nanotechnol. 2022, 17, 1136–1146. 10.1038/s41565-022-01193-2. 36163504

[ref26] Samineni L.; Acharya B.; Behera H.; Oh H.; Kumar M.; Chowdhury R. Protein engineering of pores for separation, sensing, and sequencing. Cell. Syst. 2023, 14 (8), 676–691. 10.1016/j.cels.2023.07.004. 37591205

[ref27] Schmid S.; Dekker C. Nanopores: a versatile tool to study protein dynamics. Essays Biochem. 2021, 65 (1), 93–107. 10.1042/EBC20200020. 33296461

[ref28] Chingarande R. G.; Tian K.; Kuang Y.; Sarangee A.; Hou C.; Ma E.; Ren J.; Hawkins S.; Kim J.; Adelstein R.; Chen S.; Gillis K. D.; Gu L.-Q. Real-time label-free detection of dynamic aptamer-small molecule interactions using a nanopore nucleic acid conformational sensor. Proc. Natl. Acad. Sci. U. S. A. 2023, 120 (24), e2108118120 10.1073/pnas.2108118120. 37276386 PMC10268594

[ref29] Zhang X.; Galenkamp N. S.; van der Heide N. J.; Moreno J.; Maglia G.; Kjems J. Specific Detection of Proteins by a Nanobody-Functionalized Nanopore Sensor. ACS Nano 2023, 17 (10), 9167–9177. 10.1021/acsnano.2c12733. 37127291 PMC10184537

[ref30] Jeong K.-B.; Ryu M.; Kim J.-S.; Kim M.; Yoo J.; Chung M.; Oh S.; Jo G.; Lee S.-G.; Kim H. M.; Lee M.-K.; Chi S.-W. Single-molecule fingerprinting of protein-drug interaction using a funneled biological nanopore. Nat. Commun. 2023, 14 (1), 1461 10.1038/s41467-023-37098-4. 37015934 PMC10073129

[ref31] Kang X.; Wu C.; Alibakhshi M. A.; Liu X.; Yu L.; Walt D. R.; Wanunu M. Nanopore-Based Fingerprint Immunoassay Based on Rolling Circle Amplification and DNA Fragmentation. ACS Nano 2023, 17 (6), 5412–5420. 10.1021/acsnano.2c09889. 36877993 PMC10629239

[ref32] Ying Y. L.; Yu R. J.; Hu Y. X.; Gao R.; Long Y. T. Single antibody-antigen interactions monitored via transient ionic current recording using nanopore sensors. Chem. Commun. (Camb) 2017, 53 (61), 8620–8623. 10.1039/C7CC03927A. 28721409

[ref33] Varongchayakul N.; Song J.; Meller A.; Grinstaff M. W. Single-Molecule Protein Sensing in a Nanopore: a Tutorial. Chem. Soc. Rev. 2018, 47 (23), 8512–8524. 10.1039/C8CS00106E. 30328860 PMC6309966

[ref34] Li F.; Fahie M. A.; Gilliam K. M.; Pham R.; Chen M. Mapping the conformational energy landscape of Abl kinase using ClyA nanopore tweezers. Nat. Commun. 2022, 13 (1), 3541 10.1038/s41467-022-31215-5. 35725977 PMC9209526

[ref35] Cardozo N.; Zhang K.; Doroschak K.; Nguyen A.; Siddiqui Z.; Bogard N.; Strauss K.; Ceze L.; Nivala J. Multiplexed direct detection of barcoded protein reporters on a nanopore array. Nat. Biotechnol. 2022, 40 (1), 42–46. 10.1038/s41587-021-01002-6. 34385692 PMC8766897

[ref36] Fahie M. A.; Yang B.; Mullis M.; Holden M. A.; Chen M. Selective Detection of Protein Homologues in Serum Using an OmpG Nanopore. Anal. Chem. 2015, 87 (21), 11143–11149. 10.1021/acs.analchem.5b03350. 26451707 PMC5065927

[ref37] Yu J.; Cao C.; Long Y. T. Selective and Sensitive Detection of Methylcytosine by Aerolysin Nanopore under Serum Condition. Anal. Chem. 2017, 89 (21), 11685–11689. 10.1021/acs.analchem.7b03133. 28988479

[ref38] Galenkamp N. S.; Soskine M.; Hermans J.; Wloka C.; Maglia G. Direct electrical quantification of glucose and asparagine from bodily fluids using nanopores. Nat. Commun. 2018, 9 (1), 4085 10.1038/s41467-018-06534-1. 30291230 PMC6173770

[ref39] Thakur A. K.; Movileanu L. Real-Time Measurement of Protein-Protein Interactions at Single-Molecule Resolution using a Biological Nanopore. Nat. Biotechnol. 2019, 37 (1), 96–101. 10.1038/nbt.4316. PMC6557705 30531896

[ref40] Thakur A. K.; Movileanu L. Single-Molecule Protein Detection in a Biofluid Using a Quantitative Nanopore Sensor. ACS Sens. 2019, 4 (9), 2320–2326. 10.1021/acssensors.9b00848. 31397162 PMC6764869

[ref41] Huang G.; Voorspoels A.; Versloot R. C. A.; van der Heide N. J.; Carlon E.; Willems K.; Maglia G. PlyAB Nanopores Detect Single Amino Acid Differences in Folded Haemoglobin from Blood. Angew. Chem., Int. Ed. Engl. 2022, 61 (34), e202206227 10.1002/anie.202206227. 35759385 PMC9541544

[ref42] Ahmad M.; Ha J.-H.; Mayse L. A.; Presti M. F.; Wolfe A. J.; Moody K. J.; Loh S. N.; Movileanu L. A generalizable nanopore sensor for highly specific protein detection at single-molecule precision. Nat. Commun. 2023, 14 (1), 1374 10.1038/s41467-023-36944-9. 36941245 PMC10027671

[ref43] Zhang S.; Wang Y.; Song D.; Guan S.; Zhou D.; Gong L.; Liang L.; Guan X.; Wang L. Nanopore discrimination and sensitive plasma detection of multiple natriuretic peptides: The representative biomarker of human heart failure. Biosens. Bioelectron. 2023, 231, 115299 10.1016/j.bios.2023.115299. 37054600 PMC10147535

[ref44] Greive S. J.; Bacri L.; Cressiot B.; Pelta J. Identification of Conformational Variants for Bradykinin Biomarker Peptides from a Biofluid Using a Nanopore and Machine Learning. ACS Nano 2024, 18 (1), 539–550. 10.1021/acsnano.3c08433. 38134312

[ref45] Harrington L.; Alexander L. T.; Knapp S.; Bayley H. Single-Molecule Protein Phosphorylation and Dephosphorylation by Nanopore Enzymology. ACS Nano 2019, 13 (1), 633–641. 10.1021/acsnano.8b07697. 30588793

[ref46] Sheng Y.; Zhang S.; Liu L.; Wu H. C. Measuring Enzymatic Activities with Nanopores. Chembiochem 2020, 21 (15), 2089–2097. 10.1002/cbic.202000079. 32202055

[ref47] Rodriguez-Larrea D.; Bayley H. Protein co-translocational unfolding depends on the direction of pulling. Nat. Commun. 2014, 5, 4841 10.1038/ncomms5841. 25197784 PMC4164780

[ref48] Yu L.; Kang X.; Li F.; Mehrafrooz B.; Makhamreh A.; Fallahi A.; Foster J. C.; Aksimentiev A.; Chen M.; Wanunu M. Unidirectional single-file transport of full-length proteins through a nanopore. Nat. Biotechnol. 2023, 41 (8), 1130–1139. 10.1038/s41587-022-01598-3. 36624148 PMC10329728

[ref49] Ensslen T.; Sarthak K.; Aksimentiev A.; Behrends J. C. Resolving Isomeric Posttranslational Modifications Using a Biological Nanopore as a Sensor of Molecular Shape. J. Am. Chem. Soc. 2022, 144 (35), 16060–16068. 10.1021/jacs.2c06211. 36007197

[ref50] Versloot R. C. A.; Lucas F. L. R.; Yakovlieva L.; Tadema M. J.; Zhang Y.; Wood T. M.; Martin N. I.; Marrink S. J.; Walvoort M. T. C.; Maglia G. Quantification of Protein Glycosylation Using Nanopores. Nano Lett. 2022, 22 (13), 5357–5364. 10.1021/acs.nanolett.2c01338. 35766994 PMC9284675

[ref51] Nova I. C.; Ritmejeris J.; Brinkerhoff H.; Koenig T. J. R.; Gundlach J. H.; Dekker C. Detection of phosphorylation post-translational modifications along single peptides with nanopores. Nat. Biotechnol. 2024, 42 (5), 710–714. 10.1038/s41587-023-01839-z. 37386295 PMC11189593

[ref52] Stierlen A.; Greive S. J.; Bacri L.; Manivet P.; Cressiot B.; Pelta J. Nanopore Discrimination of Coagulation Biomarker Derivatives and Characterization of a Post-Translational Modification. ACS Cent. Sci. 2023, 9 (2), 228–238. 10.1021/acscentsci.2c01256. 36844502 PMC9951287

[ref53] Martin-Baniandres P.; Lan W. H.; Board S.; Romero-Ruiz M.; Garcia-Manyes S.; Qing Y.; Bayley H. Enzyme-less nanopore detection of post-translational modifications within long polypeptides. Nat. Nanotechnol. 2023, 18 (11), 1335–1340. 10.1038/s41565-023-01462-8. 37500774 PMC10656283

[ref54] Ratinho L.; Bacri L.; Thiebot B.; Cressiot B.; Pelta J. Identification and Detection of a Peptide Biomarker and Its Enantiomer by Nanopore. ACS Cent. Sci. 2024, 10 (6), 1167–1178. 10.1021/acscentsci.4c00020. 38947203 PMC11212137

[ref55] Luchian T.; Park Y.; Asandei A.; Schiopu I.; Mereuta L.; Apetrei A. Nanoscale Probing of Informational Polymers with Nanopores. Applications to Amyloidogenic Fragments, Peptides, and DNA-PNA Hybrids. Acc. Chem. Res. 2019, 52 (1), 267–276. 10.1021/acs.accounts.8b00565. 30605305

[ref56] Afshar Bakshloo M.; Kasianowicz J. J.; Pastoriza-Gallego M.; Mathé J.; Daniel R.; Piguet F.; Oukhaled A. Nanopore-Based Protein Identification. J. Am. Chem. Soc. 2022, 144 (6), 2716–2725. 10.1021/jacs.1c11758. 35120294

[ref57] Piguet F.; Ouldali H.; Pastoriza-Gallego M.; Manivet P.; Pelta J.; Oukhaled A. Identification of Single Amino Acid Differences in Uniformly Charged Homopolymeric Peptides with Aerolysin Nanopore. Nat. Commun. 2018, 9 (1), 966 10.1038/s41467-018-03418-2. 29511176 PMC5840376

[ref58] Ouldali H.; Sarthak K.; Ensslen T.; Piguet F.; Manivet P.; Pelta J.; Behrends J. C.; Aksimentiev A.; Oukhaled A. Electrical recognition of the twenty proteinogenic amino acids using an aerolysin nanopore. Nat. Biotechnol. 2020, 38 (2), 176–181. 10.1038/s41587-019-0345-2. 31844293 PMC7008938

[ref59] Brinkerhoff H.; Kang A. S. W.; Liu J.; Aksimentiev A.; Dekker C. Multiple rereads of single proteins at single-amino acid resolution using nanopores. Science 2021, 374, 1509–1513. 10.1126/science.abl4381. 34735217 PMC8811723

[ref60] Colquhoun D.; Sigworth F. J. Fitting and statistical analysis of single-channel records. In Single-channel recording; 2nd ed.; Sackmann B.; Neher E., Ed.; Plenum Press: New York, 1995; pp 483–587.

[ref61] Couoh-Cardel S.; Hsueh Y.-C.; Wilkens S.; Movileanu L. Yeast V-ATPase Proteolipid Ring Acts as a Large-conductance Transmembrane Protein Pore. Sci. Rep. 2016, 6, 24774 10.1038/srep24774. 27098228 PMC4838861

[ref62] Schreiber G.; Haran G.; Zhou H. X. Fundamental aspects of protein-protein association kinetics. Chem. Rev. 2009, 109 (3), 839–860. 10.1021/cr800373w. 19196002 PMC2880639

[ref63] Weeramange C. J.; Fairlamb M. S.; Singh D.; Fenton A. W.; Swint-Kruse L. The strengths and limitations of using biolayer interferometry to monitor equilibrium titrations of biomolecules. Protein Sci. 2020, 29 (4), 1018–1034. 10.1002/pro.3827. 31943488 PMC7096710

[ref64] Wolfe A. J.; Si W.; Zhang Z.; Blanden A. R.; Hsueh Y. C.; Gugel J. F.; Pham B.; Chen M.; Loh S. N.; Rozovsky S.; Aksimentiev A.; Movileanu L. Quantification of membrane protein-detergent complex interactions. J. Phys. Chem. B 2017, 121 (44), 10228–10241. 10.1021/acs.jpcb.7b08045. 29035562 PMC5680101

[ref65] Wolfe A. J.; Hsueh Y. C.; Blanden A. R.; Mohammad M. M.; Pham B.; Thakur A. K.; Loh S. N.; Chen M.; Movileanu L. Interrogating Detergent Desolvation of Nanopore-Forming Proteins by Fluorescence Polarization Spectroscopy. Anal. Chem. 2017, 89 (15), 8013–8020. 10.1021/acs.analchem.7b01339. 28650154 PMC5558884

[ref66] Patel A.; Dharmarajan V.; Cosgrove M. S. Structure of WDR5 bound to mixed lineage leukemia protein-1 peptide. J. Biol. Chem. 2008, 283 (47), 32158–32161. 10.1074/jbc.C800164200. 18829459

[ref67] Smith A. M.; Lee A. A.; Perkin S. The Electrostatic Screening Length in Concentrated Electrolytes Increases with Concentration. J. Phys. Chem. Lett. 2016, 7 (12), 2157–63. 10.1021/acs.jpclett.6b00867. 27216986

[ref68] Buckle A. M.; Schreiber G.; Fersht A. R. Protein-protein recognition: crystal structural analysis of a barnase-barstar complex at 2.0-A resolution. Biochemistry 1994, 33 (30), 8878–8889. 10.1021/bi00196a004. 8043575

[ref69] Lee L. P.; Tidor B. Optimization of binding electrostatics: charge complementarity in the barnase-barstar protein complex. Protein Sci. 2001, 10 (2), 362–77. 10.1110/ps.40001. 11266622 PMC2373948

[ref70] Schreiber G.; Fersht A. R. Interaction of barnase with its polypeptide inhibitor barstar studied by protein engineering. Biochemistry 1993, 32 (19), 5145–5150. 10.1021/bi00070a025. 8494892

[ref71] Debye P.; Hückel E. Zur Theorie der Elektrolyte. Phys. Z. 1923, 24, 185–206.

[ref72] Robinson R. A.; Stokes R. H. Electrolyte Solutions; Butterworths: London, 1959.

[ref73] Schreiber G.; Fersht A. R. Rapid, electrostatically assisted association of proteins. Nat. Struct. Biol. 1996, 3 (5), 427–31. 10.1038/nsb0596-427. 8612072

[ref74] Muthukumar M. 50th Anniversary Perspective: A Perspective on Polyelectrolyte Solutions. Macromolecules 2017, 50 (24), 9528–9560. 10.1021/acs.macromol.7b01929. 29296029 PMC5746850

[ref75] Curtis R. A.; Ulrich J.; Montaser A.; Prausnitz J. M.; Blanch H. W. Protein-protein interactions in concentrated electrolyte solutions. Biotechnol. Bioeng. 2002, 79 (4), 367–380. 10.1002/bit.10342. 12115400

[ref76] Harrington L.; Cheley S.; Alexander L. T.; Knapp S.; Bayley H. Stochastic detection of Pim protein kinases reveals electrostatically enhanced association of a peptide substrate. Proc. Natl. Acad. Sci. U. S. A. 2013, 110 (47), E4417–E4426. 10.1073/pnas.1312739110. 24194548 PMC3839778

[ref77] Dharmarajan V.; Lee J. H.; Patel A.; Skalnik D. G.; Cosgrove M. S. Structural basis for WDR5 interaction (Win) motif recognition in human SET1 family histone methyltransferases. J. Biol. Chem. 2012, 287 (33), 27275–27289. 10.1074/jbc.M112.364125. 22665483 PMC3431640

[ref78] Patel A.; Vought V. E.; Dharmarajan V.; Cosgrove M. S. A conserved arginine-containing motif crucial for the assembly and enzymatic activity of the mixed lineage leukemia protein-1 core complex. J. Biol. Chem. 2008, 283 (47), 32162–32175. 10.1074/jbc.M806317200. 18829457

[ref79] Vedadi M.; Blazer L.; Eram M. S.; Barsyte-Lovejoy D.; Arrowsmith C. H.; Hajian T. Targeting human SET1/MLL family of proteins. Protein Sci. 2017, 26 (4), 662–676. 10.1002/pro.3129. 28160335 PMC5368065

[ref80] Sha L.; Ayoub A.; Cho U. S.; Dou Y. Insights on the regulation of the MLL/SET1 family histone methyltransferases. Biochim. Biophys. Acta Gene. Regul. Mech. 2020, 1863 (7), 194561 10.1016/j.bbagrm.2020.194561. 32304759 PMC7236755

[ref81] Antunes E. T. B.; Ottersbach K. The MLL/SET family and haematopoiesis. Biochim. Biophys. Acta Gene Regul. Mech. 2020, 1863 (8), 194579 10.1016/j.bbagrm.2020.194579. 32389825 PMC7294230

[ref82] Zhang P.; Lee H.; Brunzelle J. S.; Couture J. F. The plasticity of WDR5 peptide-binding cleft enables the binding of the SET1 family of histone methyltransferases. Nucleic Acids Res. 2012, 40 (9), 4237–4246. 10.1093/nar/gkr1235. 22266653 PMC3351189

[ref83] Mayse L. A.; Imran A.; Larimi M. G.; Cosgrove M. S.; Wolfe A. J.; Movileanu L. Disentangling the recognition complexity of a protein hub using a nanopore. Nat. Commun. 2022, 13 (1), 978 10.1038/s41467-022-28465-8. 35190547 PMC8861093

[ref84] Guarnaccia A. D.; Rose K. L.; Wang J.; Zhao B.; Popay T. M.; Wang C. E.; Guerrazzi K.; Hill S.; Woodley C. M.; Hansen T. J.; Lorey S. L.; Shaw J. G.; Payne W. G.; Weissmiller A. M.; Olejniczak E. T.; Fesik S. W.; Liu Q.; Tansey W. P. Impact of WIN site inhibitor on the WDR5 interactome. Cell Rep. 2021, 34 (3), 108636 10.1016/j.celrep.2020.108636. 33472061 PMC7871196

[ref85] Imran A.; Moyer B. S.; Kalina D.; Duncan T. M.; Moody K. J.; Wolfe A. J.; Cosgrove M. S.; Movileanu L. Convergent Alterations of a Protein Hub Produce Divergent Effects Within a Binding Site. ACS Chem. Biol. 2022, 17 (6), 1586–1597. 10.1021/acschembio.2c00273. 35613319 PMC9207812

[ref86] Ali A.; Veeranki S. N.; Tyagi S. A SET-domain-independent role of WRAD complex in cell-cycle regulatory function of mixed lineage leukemia. Nucleic Acids Res. 2014, 42 (12), 7611–24. 10.1093/nar/gku458. 24880690 PMC4081079

[ref87] Zhao J.; Chen W.; Pan Y.; Zhang Y.; Sun H.; Wang H.; Yang F.; Liu Y.; Shen N.; Zhang X.; Mo X.; Zang J. Structural insights into the recognition of histone H3Q5 serotonylation by WDR5. Sci. Adv. 2021, 7 (25), eabf4291 10.1126/sciadv.abf4291. 34144982 PMC8213231

[ref88] Kalkat M.; Resetca D.; Lourenco C.; Chan P.-K.; Wei Y.; Shiah Y.-J.; Vitkin N.; Tong Y.; Sunnerhagen M.; Done S. J.; Boutros P. C.; Raught B.; Penn L. Z. MYC Protein Interactome Profiling Reveals Functionally Distinct Regions that Cooperate to Drive Tumorigenesis. Mol. Cell 2018, 72 (5), 836–848.e7. 10.1016/j.molcel.2018.09.031. 30415952

[ref89] Conacci-Sorrell M.; McFerrin L.; Eisenman R. N. An overview of MYC and its interactome. Cold Spring Harb. Perspect. Med. 2014, 4 (1), a014357 10.1101/cshperspect.a014357. 24384812 PMC3869278

[ref90] Lourenco C.; Resetca D.; Redel C.; Lin P.; MacDonald A. S.; Ciaccio R.; Kenney T. M. G.; Wei Y.; Andrews D. W.; Sunnerhagen M.; Arrowsmith C. H.; Raught B.; Penn L. Z. MYC protein interactors in gene transcription and cancer. Nat. Rev. Cancer 2021, 21 (9), 579–591. 10.1038/s41568-021-00367-9. 34188192

[ref91] Imran A.; Moyer B. S.; Wolfe A. J.; Cosgrove M. S.; Makarov D. E.; Movileanu L. Interplay of Affinity and Surface Tethering in Protein Recognition. J. Phys. Chem. Lett. 2022, 13 (18), 4021–4028. 10.1021/acs.jpclett.2c00621. 35485934 PMC9106920

[ref92] Harrington L.; Alexander L. T.; Knapp S.; Bayley H. Pim Kinase Inhibitors Evaluated with a Single-Molecule Engineered Nanopore Sensor. Angew. Chem., Int. Ed. Engl. 2015, 54 (28), 8154–9. 10.1002/anie.201503141. 26058458

[ref93] Wolf E.; Eilers M. Targeting MYC Proteins for Tumor Therapy. Annu. Rev. Cancer Biol. 2020, 4, 61–75. 10.1146/annurev-cancerbio-030518-055826.

[ref94] Mayse L. A.; Imran A.; Wang Y.; Ahmad M.; Oot R. A.; Wilkens S.; Movileanu L. Evaluation of Nanopore Sensor Design Using Electrical and Optical Analyses. ACS Nano 2023, 17 (11), 10857–10871. 10.1021/acsnano.3c02532. 37261404 PMC10278182

[ref95] Mohammad M. M.; Howard K. R.; Movileanu L. Redesign of a plugged beta-barrel membrane protein. J. Biol. Chem. 2011, 286 (10), 8000–8013. 10.1074/jbc.M110.197723. 21189254 PMC3048687

[ref96] Mohammad M. M.; Iyer R.; Howard K. R.; McPike M. P.; Borer P. N.; Movileanu L. Engineering a Rigid Protein Tunnel for Biomolecular Detection. J. Am. Chem. Soc. 2012, 134 (22), 9521–9531. 10.1021/ja3043646. 22577864 PMC3415594

[ref97] Larimi M. G.; Mayse L. A.; Movileanu L. Interactions of a Polypeptide with a Protein Nanopore Under Crowding Conditions. ACS Nano 2019, 13 (4), 4469–4477. 10.1021/acsnano.9b00008. 30925041 PMC6482057

[ref98] Sharma S.; Wilkens S. Biolayer interferometry of lipid nanodisc-reconstituted yeast vacuolar H(+) -ATPase. Protein Sci. 2017, 26 (5), 1070–1079. 10.1002/pro.3143. 28241399 PMC5405429

[ref99] Imran A.; Moyer B. S.; Canning A. J.; Kalina D.; Duncan T. M.; Moody K. J.; Wolfe A. J.; Cosgrove M. S.; Movileanu L. Kinetics of the multitasking high-affinity Win binding site of WDR5 in restricted and unrestricted conditions. Biochem. J. 2021, 478 (11), 2145–2161. 10.1042/BCJ20210253. 34032265 PMC8214142

