# Supplementary material for: Real-Time Measurement of a Weak Interaction of a Transcription Factor Motif with a Protein Hub at Single-Molecule Precision
Source: ACS Nano. 2024 Jul 25;18(31):20468–81. doi: 10.1021/acsnano.4c04857 (PMC11308778; doi:10.1021/acsnano.4c04857)
Supplement: Supplementary file 1 — nn4c04857_si_001.pdf [file nn4c04857_si_001.pdf]

SUPPORTING INFORMATION FILE

Real-Time Measurement of a Weak Interaction of a Transcription Factor Motif With a Protein Hub at Single-Molecule Precision

Lauren A. Mayse,[1,2] Yazheng Wang,[1,2] Mohammad Ahmad,[1] and Liviu Movileanu[1,2,3,4&]

[1]*Department of Physics, Syracuse University, 201 Physics Building, Syracuse, New York 13244, USA*

[2]*Department of Biomedical and Chemical Engineering, Syracuse University, 329 Link Hall, Syracuse, New York 13244, USA*

[3]*Department of Biology, Syracuse University, 114 Life Sciences Complex, Syracuse, New York 13244, USA*

[4]*The BioInspired Institute, Syracuse University, Syracuse, New York 13244, USA*

[&]The corresponding author's contact information:
Liviu Movileanu
E-mail: lmovilea@syr.edu

Table S1. List of hydrogen bonds, hydrophobic contacts, and ionic bonds at the MYC_{WBM}–WDR5 interaction interface. The blue and red residues in each interaction pair belong to MYC_{WBM} and WDR5, respectively. These interactions were determined using protein interactions calculator (PIC)[1] and previously published co-crystallization data (PDB: 4Y7R).[2] The cut-off distance for identifying these hydrogen bonds was 4.0 Å. The cut-off distances for hydrophobic and ionic interactions were 5.0 and 6.0 Å, respectively.

Peptide	Hydrogen bonds	Hydrophobic contacts	Ionic bonds
**MYC_{WBM}**	V9 - N225	I7 - Y228	E4 - K245
**QEDEEEIDVVSVE**	V10 - N225	I7 - L240	E5 - K247
	D8 - N225	I7 - L249	E6 - K247
	E6 - K250	V9 - F266	D8 - K250
	I7 - Q289	V9 - V268	E5 - K272
	S11- N225	V10 - F266	
		V10 - V268	

Table S2. The open-state currents of tFhuA and MYC_{WBM}tFhuA. The open-state current, I_0, was measured at a transmembrane potential of -20 mV.

Nanopore Sensor	I_0 (pA)
**tFhuA**	-30 ± 3
**MYC_{WBM}tFhuA**	-24 ± 2

Values are mean ± s.d. obtained from n = 8 independently reconstituted nanopores for tFhuA and MYC_{WBM}tFhuA. The other experimental conditions were the same as those stated in the **Experimental section.**

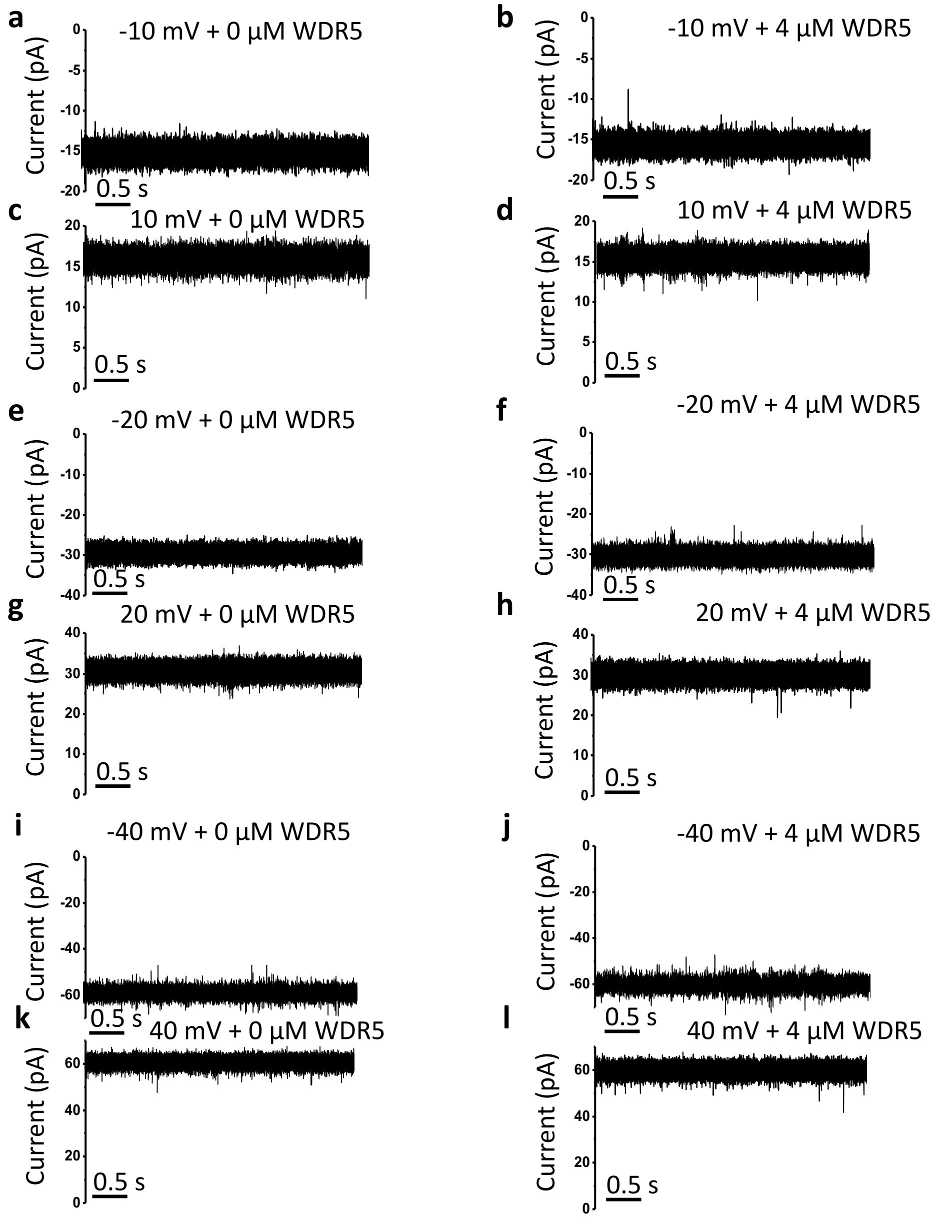

Figure S1. Representative single-channel electrical traces acquired with the unmodified tFhuA nanopore. These traces were low-pass filtered using an 8-pole Bessel filter at 1 kHz. **(a)** A representative single-channel electrical trace was acquired with a tFhuA[3, 4] nanopore without WDR5. The applied transmembrane potential was −10 mV. **(b)** Representative single-channel electrical trace of (a) but in the presence of 4 µM WDR5 added to the *cis* compartment. **(c)** Representative single-channel electrical trace as in (a) but recorded at an applied transmembrane potential of +10 mV. **(d)** Representative single-channel electrical trace as in (c), but in the presence of 4 µM WDR5 added to the *cis* compartment. **(e)** Representative single-channel electrical trace as in (a) but recorded at an applied transmembrane potential of -20 mV. **(f)** Representative single-channel electrical trace as in (e), but in the presence of 4 µM WDR5 added to the *cis* compartment. **(g)** Representative single-channel electrical trace as in (a) but recorded at an applied transmembrane potential of +20 mV.

(h) Representative single-channel electrical trace as in (g), but in the presence of 4 μM WDR5 added to the *cis* compartment. **(i)** Representative single-channel electrical trace as in (a) but recorded at an applied transmembrane potential of -40 mV. **(j)** Representative single-channel electrical trace as in (i), but in the presence of 4 μM WDR5 added to the *cis* compartment. **(k)** Representative single-channel electrical trace as in (a) but recorded at an applied transmembrane potential of +40 mV. **(l)** Representative single-channel electrical trace as in (k), but in the presence of 4 μM WDR5 added to the *cis* compartment. These traces were replicated in at least $n = 3$ independent single-molecule reconstitutions. The other experimental conditions were the same as those in the **Experimental section**.

Table S3. The O_{on} and O_{off} current substates for all [WDR5] values. I_{on} and I_{off} are the single-channel currents corresponding to O_{on} and O_{off} substates, respectively (**Figure 2**). They were directly measured at a transmembrane potential of -20 mV and in 300 mM KCl, 20 mM Tris-HCl, 1 mM TCEP, and pH 7.5.

[WDR5] (μM)	I_{on} (pA)	I_{off} (pA)
2	23 ± 2	14 ± 2
4	22 ± 2	14 ± 3
7.8	22 ± 1	13 ± 1
11.4	21 ± 2	15 ± 2
18.1	22 ± 1	13 ± 1

Values are mean ± s.d. obtained from $n = 7$ independently reconstituted $MYC_{WBM}tFhuA$ nanopores. The other experimental conditions were the same as those stated in the **Experimental section**.

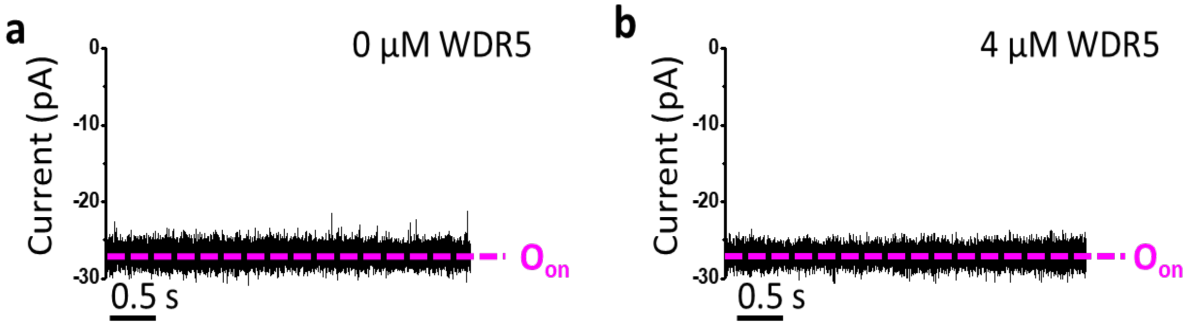

Figure S2. Representative single-channel electrical traces were acquired with a single adaptor-free $MYC_{WBM}tFhuA$ nanopore sensor. (a) A representative single-channel electrical trace was acquired with an $MYC_{WBM}tFhuA$ nanopore without the peptide adaptor and WDR5. The applied transmembrane potential was −20 mV. **(b)** A representative single-channel electrical trace of $MYC_{WBM}tFhuA$ without the peptide adaptor in the presence of 4 μM WDR5 added to the *cis* compartment. No current blockades were noted. These single-channel electrical traces were low-pass filtered using an 8-pole Bessel filter at 1 kHz. These traces were replicated in $n = 3$ independent single-molecule reconstitutions. The other experimental conditions were the same as those stated in the **Experimental section**.

Table S4. Values of the WDR5-unbound (τ_{on}) and WDR5-bound durations (τ_{off}) using a MYC_{WBM}tFhuA nanopore sensor at various [WDR5] values. τ_{on} are mean values of the single-exponential distributions of WDR5-unbound duration histograms. τ_{off} values are the mean values of the single-exponential distributions of WDR5-bound duration histograms. The maximum likelihood method[5-7] and logarithm likelihood ratio (LLR)[8-10] tests were used for all fittings to determine the best model of probability distribution function (PDF; **Experimental section**).

n	**[WDR5] (μM)**	**τ_{on} (s)**	**τ_{off} (s)**
4	2	3.4 ± 0.5	0.042 ± 0.005
4	4	1.7 ± 0.3	0.041 ± 0.005
3	7.8	0.89 ± 0.18	0.036 ± 0.004
4	11.4	0.65 ± 0.06	0.038 ± 0.004
3	18.1	0.43 ± 0.06	0.038 ± 0.004

Values indicate mean ± s.d. *n* is the number of independently reconstituted nanopores. The other experimental conditions were the same as those stated in the **Experimental section**.

Table S5. The association rate constants (k_{on}) for the MYC_{WBM}-WDR5 interaction. The k_{on} values were determined as the slope of the linear fit in **Figure 3a.** The association rate constants are also confirmed for each [WDR5] value using the equation $k_{on} = 1/(\tau_{on}[\text{WDR5}])$. The τ_{on} values are provided in **Supplementary Table S4**.

n	**[WDR5] (μM)**	**k_{on} (10^5 $M^{-1}s^{-1}$)**
NA	from fitting	1.4 ± 0.1
4	2	1.5 ± 0.2
5	4	1.6 ± 0.3
5	7.8	1.5 ± 0.3
4	11.4	1.4 ± 0.1
4	18.1	1.3 ± 0.2

Values indicate mean ± s.e.m. from fitted data and mean ± s.d. for calculated rate constants at specific [WDR5] values. *n* is the number of independently reconstituted nanopores. NA denotes not applicable. The other experimental conditions were the same as those stated in the **Experimental section**.

Table S6. The dissociation rate constants (k_{off}) for the MYC_{WBM}-WDR5 interaction. The k_{off} was determined as the y-intercept of the horizontal line fit with the vertical axis in **Figure 3b.** The k_{off} was confirmed for each [WDR5] value using $k_{off} = 1/\tau_{off}$. The τ_{off} values are provided in **Supplementary Table S4**.

n	**[WDR5] (μM)**	**k_{off} (s^{-1})**
NA	from fitting	26 ± 1
4	2	25 ± 3
5	4	25 ± 3
5	7.8	28 ± 3
4	11.4	27 ± 2
4	18.1	26 ± 3

Values indicate mean ± s.e.m. from fitted data and mean ± s.d. for calculated rate constants at specific [WDR5] values. *n* is the number of independently reconstituted nanopores. NA indicates not applicable. The other experimental conditions were the same as those stated in the **Experimental section**.

Table S7. The equilibrium dissociation constant, K_D, for the MYC_{WBM}-WDR5 interaction. The K_D values were determined using $K_D = k_{off}/k_{on}$. The respective k_{on} and k_{off} values are in **Supplementary Tables S5-S6,** respectively.

	**K_D (μM)**
**MYC_{WBM}tFhuA-WDR5**	200 ± 21

Values indicate mean ± s.d. *n* =10*, n* is the number of independently reconstituted nanopores. Single-channel electrical recordings were performed using a solution containing 300 mM KCl, 20 mM Tris-HCl, 1 mM TCEP, and pH 7.5. The other experimental conditions were the same as those stated in the **Experimental section**.

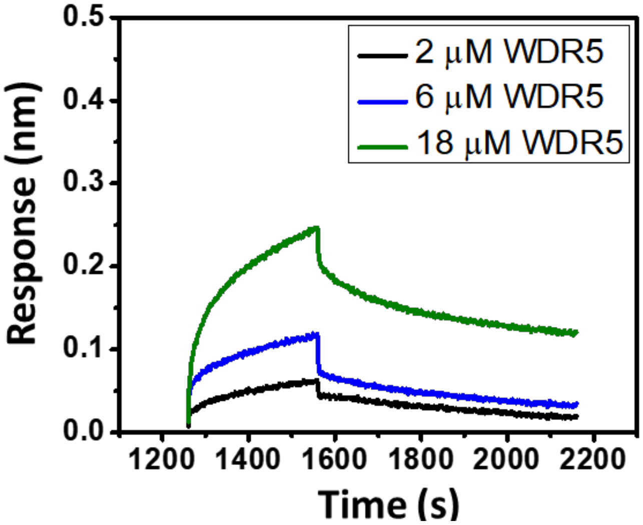

Figure S3. ND-BLI sensorgrams of the MYC$_{WBM}$tFhuA-WDR5 interaction. A serial dilution of WDR5 ranging from 2 μM to 18 μM was used to obtain individual binding curves. **(a)** ND-BLI sensorgrams show a dose-dependent response following the interaction between MYC$_{WBM}$ and WDR5. MYC$_{WBM}$tFhuA was reconstituted into biotinylated ND (**Experimental section**)[11] to form the ND-MYC$_{WBM}$tFhuA complex, which was immobilized on the BLI sensor surface. 15 nM biotin-tagged ND-MYC$_{WBM}$tFhuA was loaded onto streptavidin (SA) sensors for 5 minutes. The running buffer was 300 mM KCl, 20 mM Tris-HCl, 1 mM TCEP, 1 mg/ml bovine serum albumin (BSA), and pH 7.5. These traces were replicated in n = 3 independent ND-BLI experiments.

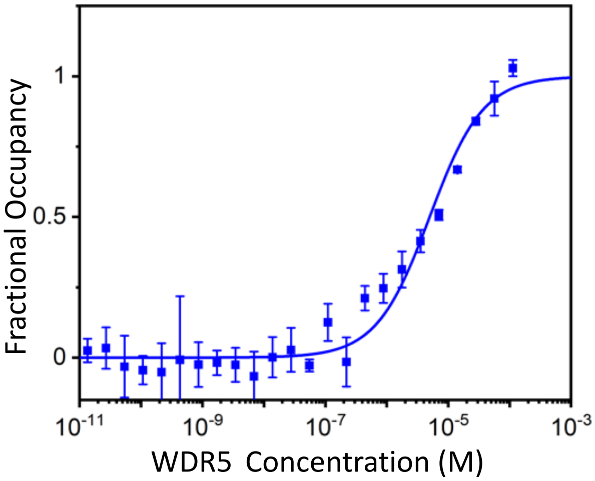

Figure S4. Steady-state fluorescence polarization (FP) anisotropy of the MYC$_{WBM}$-WDR5 interaction. The FP data was collected using a Spectramax i3 microplate reader (Molecular Devices, San Jose, CA; the **Experimental section**).[12, 13] The N terminus of the MYC$_{WBM}$ peptide was labeled using fluorescein isothiocyanate (FITC) via a hexapeptide $(GGS)_2$ linker, while the C terminus was amidated (FITC-GGSGGSQEDEEEIDVVSVE-NH). The final concentration of labeled peptide in each well was 50 nM. Triplicates were performed to obtain the WDR5 dose response. Experiments were conducted in 150 mM NaCl, 20 mM Tris-HCl, 1 mM TCEP, and pH 7.5. An equilibrium dissociation constant, K_D, of 5.0 ± 0.9 μM was identified.

Table S8. Determination of the mean activity coefficient as a function depending on tested salt concentrations. I is the ionic strength of each [KCl] value. λ_D is the Debye-Hückel screening length for each tested condition.[14] The mean activity coefficient ($f_{\pm}^{*}$) was calculated using the extended Debye-Hückel equation: $log\, f_{\pm}^{*} = \frac{A|Z_a Z_b|\sqrt{I}}{1+Ba\sqrt{I}}$, where A is a constant that depends on room temperature and B is a constant that depends on the dielectric constant of the solution.[15-17] $A = 0.5046\ dm^{3/2}\cdot mol^{-1/2}$ and $B = 3.276 \times 10^8\ dm^{1/2}\cdot mol^{-1/2}$.[18] a is the hydration diameter of K, which is 0.45×10^{-9} m.[19, 20] Z_a and Z_b are the valency numbers for K and Cl, respectively.

[KCl] (mM)	I (M)	λ_D (nm)	$\log f_{\pm}^{*}$
50	0.05	1.4	-0.08
100	0.1	0.96	-0.11
200	0.2	0.68	-0.14
300	0.3	0.56	-0.15
400	0.4	0.48	-0.17

Table S9. The unitary conductance of MYC$_{WBM}$tFhuA, as well as the O$_{on}$ and O$_{off}$ current states for various [WDR5] values and at different salt concentrations. The O$_{on}$ current, I_{on}, is the base current that corresponds to unbound events. The conductance was determined as I_{on} divided by the applied voltage and reported in nS. The O$_{off}$ current, I_{off}, corresponds to bound events. These currents are directly measured at a transmembrane potential of -20 mV.

[KCl] (mM)	Conductance (nS)	[WDR5] (µM)	I_{on} (pA)	I_{off} (pA)
50	0.26 ± 0.02	4	5 ± 3	3 ± 1
		7.8	5 ± 2	3 ± 2
		11.4	5 ± 1	2 ± 1
100	0.45 ± 0.05	4	9 ± 2	6 ± 2
		7.8	10 ± 3	7 ± 2
		11.4	9 ±1	5 ± 1
200	0.85 ± 0.10	4	17 ± 3	10 ± 2
		7.8	18 ± 2	11 ± 3
		11.4	17 ± 2	10 ± 3
300	1.3 ± 0.1	4	23 ± 2	14 ± 2
		7.8	21 ± 2	15 ± 1
		11.4	22 ± 1	14 ± 3
400	1.6 ± 0.1	4	32 ± 3	18 ± 3
		7.8	33 ± 2	20 ± 4
		11.4	32 ± 1	18 ± 2

Values are mean ± s.d. obtained from $n = 5$ independently reconstituted MYC$_{WBM}$tFhuA nanopores. The other experimental conditions were the same as those stated in the **Experimental section**.

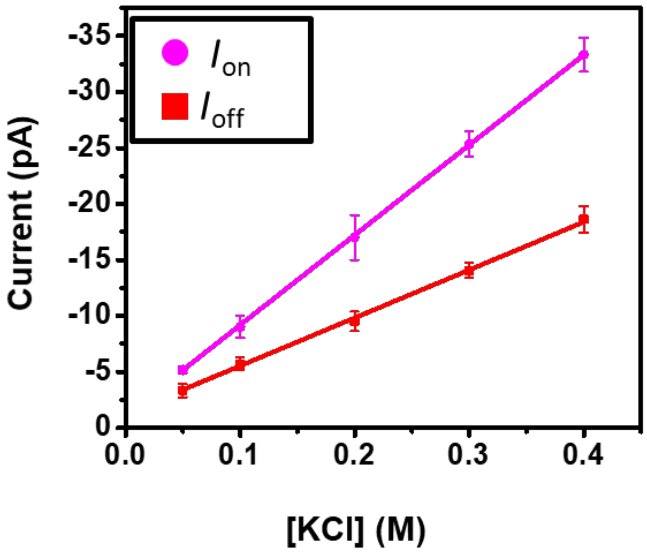

Figure S5. The unbound, I_{on}, and bound, I_{off}, current substates as a function of [KCl]. I_{on} is the current when WDR5 is not bound to MYC_{WBM}. I_{off} is the current that corresponds to bound events. The applied transmembrane potential was −20 mV. Data points represent mean ± s.d. obtained from $n = 5$ distinct experiments. The other experimental conditions were the same as those stated in the **Experimental section**.

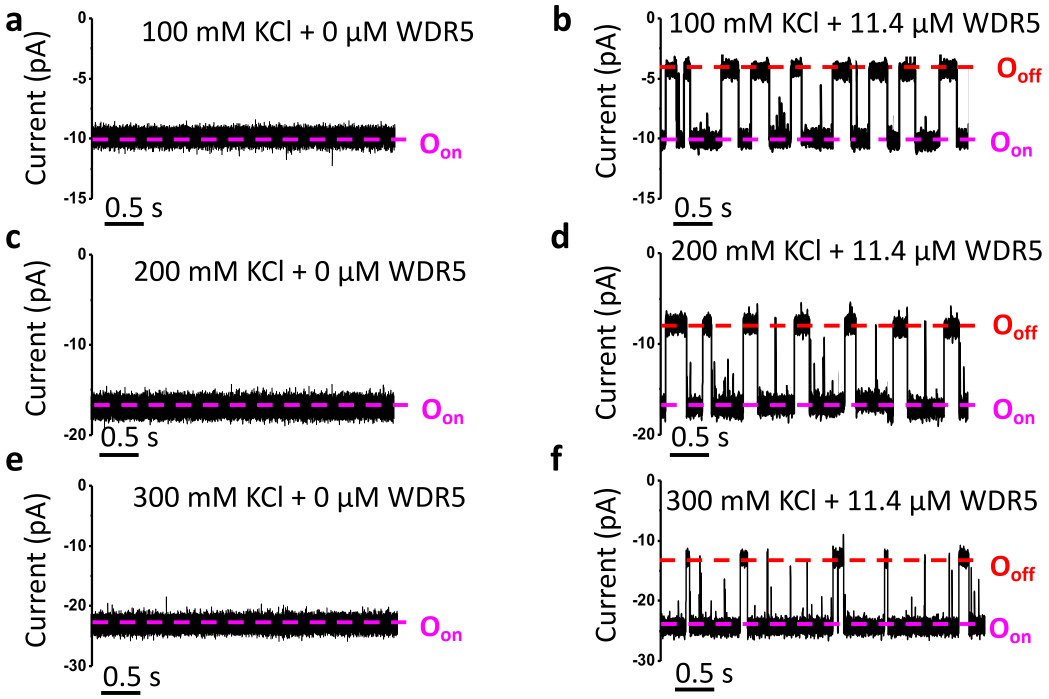

Figure S6. Representative single-channel electrical traces acquired in the presence and absence of WDR5 and at various KCl concentrations. The applied transmembrane potential was −20 mV. These single-channel electrical traces were low-pass filtered using an 8-pole Bessel filter at 1 kHz, except traces at 100 mM KCl, which were filtered at a frequency of 0.1 kHz. 11.4 µM WDR5 was added to the *cis* side. **(a)** A representative single-channel electrical trace was acquired with MYC_{WBM}tFhuA without WDR5 and tested in 100 mM KCl. **(b)** The same as (a), but in the presence of 11.4 µM WDR5. **(c)** The same as (a) but tested in 200 mM KCl. **(d)** The same as (c) but tested in the presence of 11.4 µM WDR5. **(e)** The same as (a) but tested in 300 mM KCl. **(f)** The same as (e) but tested in the presence of 11.4 µM WDR5. These traces are representative of a number $n = 4$ distinct experiments. The other experimental conditions were the same as those stated in the **Experimental section**.

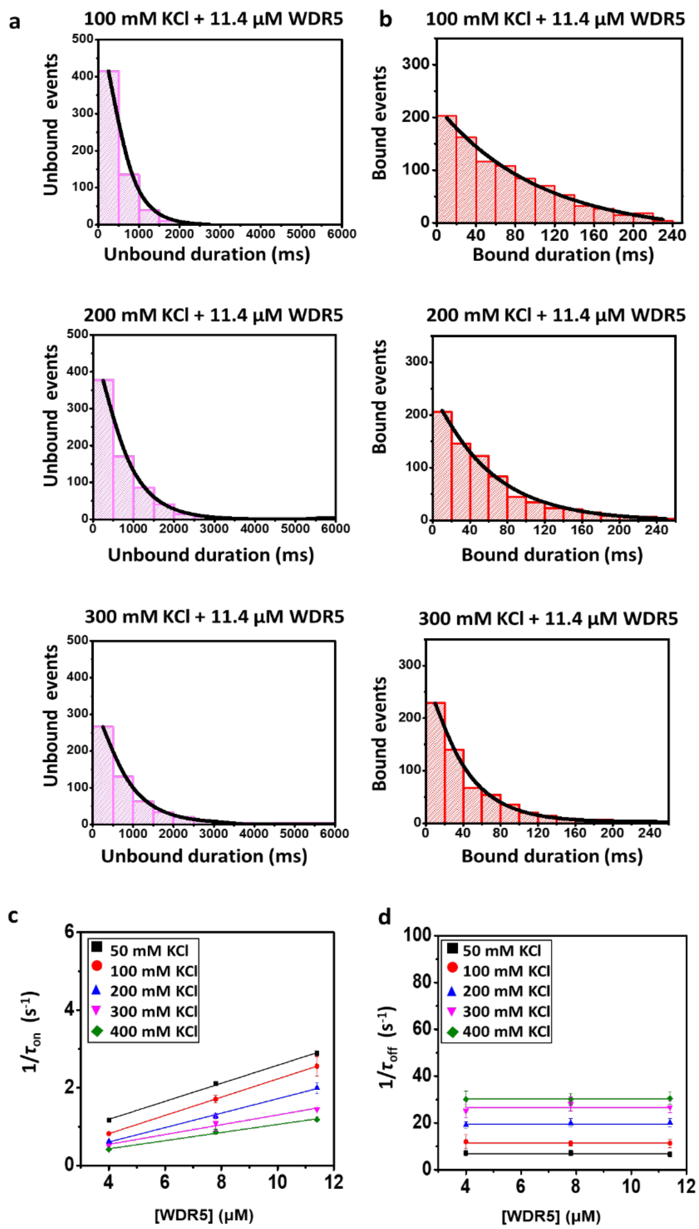

Figure S7. Event histograms of WDR5-unbound and WDR5-bound durations at different [KCl] values. This data is from the representative trace of a reconstituted nanopore (**Supplementary Figure S6**).11.4 μM WDR5 was added to the *cis* side. **(a)** Event histograms of the WDR5-unbound durations (τ_{on}). The τ_{on} values (mean ± s.e.m.) were 375 ± 20 ms (number of events: N = 875), 510 ± 31 ms (N =711), and 692 ± 70 ms (N = 550) at 100, 200, and 300 mM KCl, respectively. (**b**) Event histograms of the WDR5-bound durations (τ_{off}). The τ_{off} values (mean ± s.e.m.) were 89 ± 2 ms (number of events: N = 891), 50 ± 4 ms (N =726), and 43 ± 4 ms (N = 587) at 100, 200, and 300 mM KCl, respectively. **(c)** Dependence of $1/\tau_{on}$ on [WDR5]. **(d)** Dependence of $1/\tau_{off}$ on [WDR5]. Data points for (c) and (d) represent mean ± s.d. obtained from n = 4 distinct experiments. The other experimental conditions were the same as those stated in the **Experimental section**.

Table S10. Values of the WDR5-unbound durations (τ_{on}) and WDR5-bound durations (τ_{off}) for various [WDR5] values and at different KCl concentrations. τ_{on} values are the mean values of the single-exponential distributions of WDR5-unbound durations. τ_{off} values are the mean values of the single-exponential distributions of WDR5-bound durations. The maximum likelihood method[5-7] and logarithm likelihood ratio (LLR)[8-10] tests were utilized for all fittings to determine the best model of probability distribution function (PDF) of single-channel events (**Experimental section**).

[KCl] (mM)	[WDR5] (μM)	τ_{on} (s)	τ_{off} (s)
50	4	0.86 ± 0.05	0.14 ± 0.03
	7.8	0.47 ± 0.01	0.14 ± 0.02
	11.4	0.35 ± 0.01	0.15 ± 0.02
100	4	1.2 ± 0.1	0.087 ± 0.002
	7.8	0.59 ± 0.03	0.089 ± 0.001
	11.4	0.39 ± 0.02	0.091 ± 0.002
200	4	1.6 ± 0.1	0.052 ± 0.004
	7.8	0.78 ± 0.1	0.050 ± 0.004
	11.4	0.50 ± 0.1	0.050 ± 0.005
300	4	1.9 ± 0.1	0.038 ± 0.005
	7.8	0.9 ± 0.2	0.040 ± 0.004
	11.4	0.7 ± 0.1	0.035 ± 0.003
400	4	2.4 ± 0.1	0.033 ± 0.004
	7.8	1.1 ± 0.1	0.030 ± 0.003
	11.4	0.8 ± 0.1	0.033 ± 0.003

Values indicate mean ± s.d. $n = 4$ for all conditions shown. n is the number of independently reconstituted MYC_{WBM}tFhuA nanopores. The other experimental conditions were the same as those stated in the **Experimental section**.

Table S11. Dependence of the association rate constants (k_{on}) for the MYC$_{WBM}$-WDR5 interaction on [KCl]. k_{on} values are determined as the slope of the linear line fit in **Supplementary Figure 7c.** The association rate constant is confirmed for each [WDR5] value using the equation $k_{on} = 1/(\tau_{on}[WDR5])$. τ_{on} values are provided in **Supplementary Table S10.**

N	[KCl] (mM)	[WDR5] (μM)	$k_{on} \times 10^{-5}$ ($M^{-1}s^{-1}$)
NA	50	from fitting	2.6 ± 0.1
NA	100	from fitting	2.2 ± 0.1
NA	200	from fitting	1.7 ± 0.1
NA	300	from fitting	1.3 ± 0.1
NA	400	from fitting	1.0 ± 0.1
3	50	4	2.9 ± 0.2
		7.8	2.7 ± 0.1
		11.4	2.5 ± 0.1
4	100	4	2.1± 0.1
		7.8	2.2 ± 0.1
		11.4	2.2 ± 0.2
3	200	4	1.6 ± 0.1
		7.8	1.6 ± 0.1
		11.4	1.8 ± 0.1
5	300	4	1.3 ± 0.1
		7.8	1.4 ± 0.1
		11.4	1.3 ± 0.1
3	400	4	1.0 ± 0.1
		7.8	1.1 ± 0.1
		11.4	1.0 ± 0.1

Values are mean ± s.d. *n* is the number of independently reconstituted nanopores. NA indicates not applicable data. The other experimental conditions were the same as those stated in the **Experimental section**.

Table S12. The dissociation rate constants (k_{off}) for MYC$_{WBM}$-WDR5 interaction under different [KCl]. k_{off} values are determined as the y-intercept of the horizontal line fits with the vertical axis in **Supplementary Figure 7d.** The dissociation rate constant is confirmed for each [WDR5] value using $k_{off} = 1/(\tau_{off})$. τ_{off} values are provided in **Supplementary Table S10.**

n	**[KCl] (mM)**	**[WDR5] (µM)**	**k_{off} (s^{-1})**
NA	50	from fitting	7.0 ± 0.1
NA	100	from fitting	12 ± 1
NA	200	from fitting	21 ± 1
NA	300	from fitting	27 ± 1
NA	400	from fitting	30 ± 1
3	50	4	7.1 ± 1.4
		7.8	7.1 ± 1.1
		11.4	6.6 ± 0.9
4	100	4	12 ± 3
		7.8	11 ± 1
		11.4	11 ± 2
3	200	4	19 ± 1
		7.8	20 ± 2
		11.4	20 ± 2
5	300	4	25 ± 3
		7.8	28 ± 3
		11.4	27 ± 2
3	400	4	30 ± 3
		7.8	30 ± 3
		11.4	31 ± 3

Values indicate mean ± s.d. *n* is the number of independently reconstituted nanopores. NA indicates not applicable data. The other experimental conditions were the same as those stated in the **Experimental section**.

Table S13. The equilibrium dissociation constant, K_D, for the MYC$_{WBM}$-WDR5 complex at different [KCl]. K_D values were determined using the equation $K_D = k_{off}/k_{on}$. The respective k_{on} and k_{off} values are listed in **Supplementary Tables S11-S12,** respectively.

[KCl] (mM)	[WDR5] (µM)	K_D (µM)
50	4	24 ± 4
	7.8	26 ± 5
	11.4	26 ± 3
100	4	58 ±13
	7.8	52 ± 6
	11.4	51 ± 13
200	4	122 ± 8
	7.8	122 ± 15
	11.4	115 ± 13
300	4	192 ± 2
	7.8	197 ± 2
	11.4	203 ± 2
400	4	291 ± 4
	7.8	272 ± 3
	11.4	293 ± 3

Values were provided as mean ± s.d. n is the number of independently reconstituted nanopores. For this table, n= 3. The other experimental conditions were the same as those stated in the **Experimental section**.

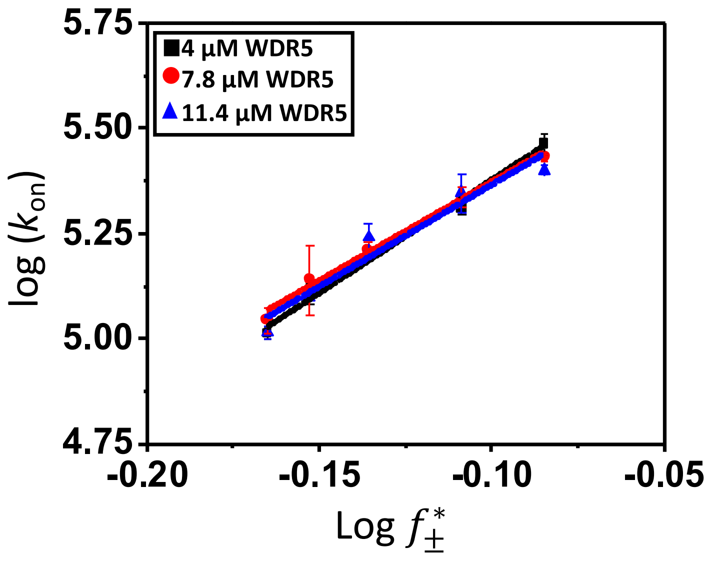

Figure S8. The association rate constant (k_{on}) is a function that depends on the mean activity coefficient. The k_{on} values are listed in **Supplementary Table S11.** The mean activity coefficients are provided in **Supplementary Table S8.** The y-intercept of this plot represents the predicted association rate constant in the absence of the electrolyte, $k_{on}(0)$. Here, $k_{on}(0) = (2.9 \pm 0.1) \times 10^5$ M^{-1}s^{-1}. Data points represent mean ± s.d. obtained from $n = 3$ distinct experiments. The other experimental conditions were the same as those stated in the **Experimental section**.

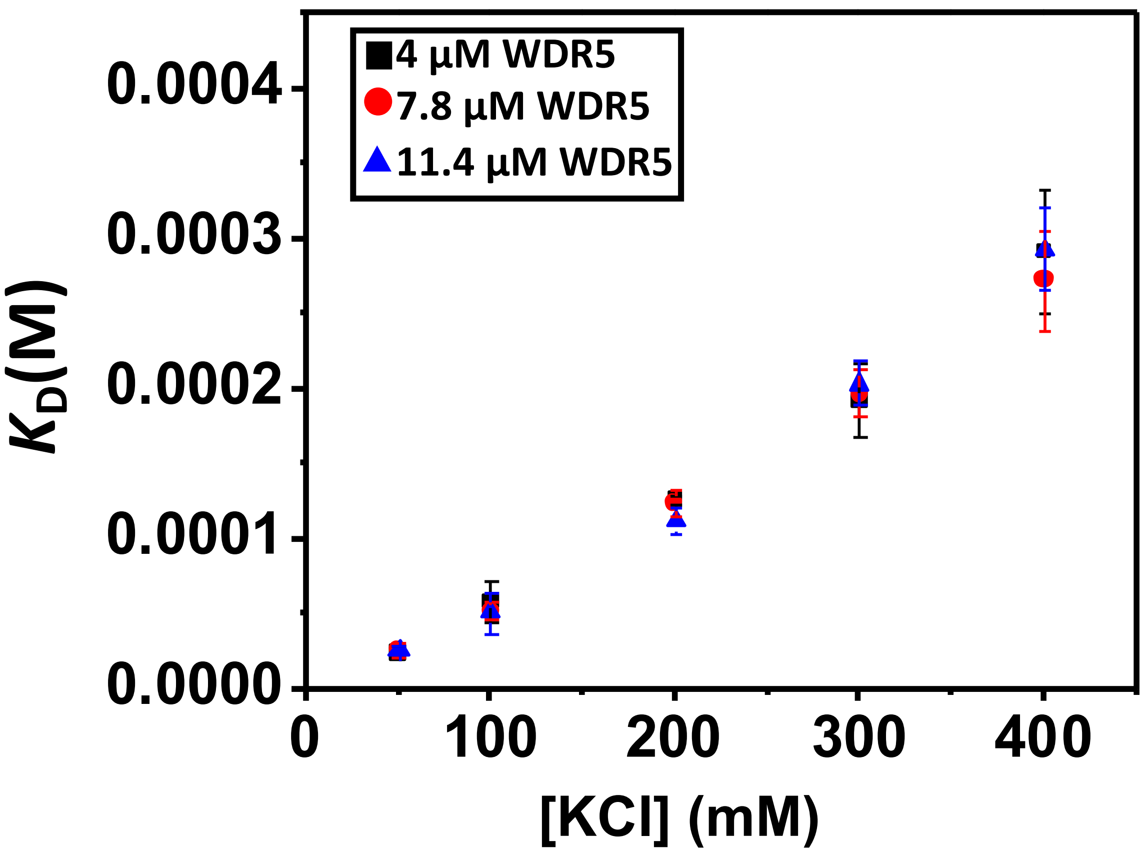

Figure S9. The equilibrium dissociation constant, K_D, as a function of [KCl]. K_D values are listed in **Supplementary Table S13**. Data points represent mean ± s.d. obtained from $n = 3$ distinct experiments. The other experimental conditions were the same as those stated in the **Experimental section**.

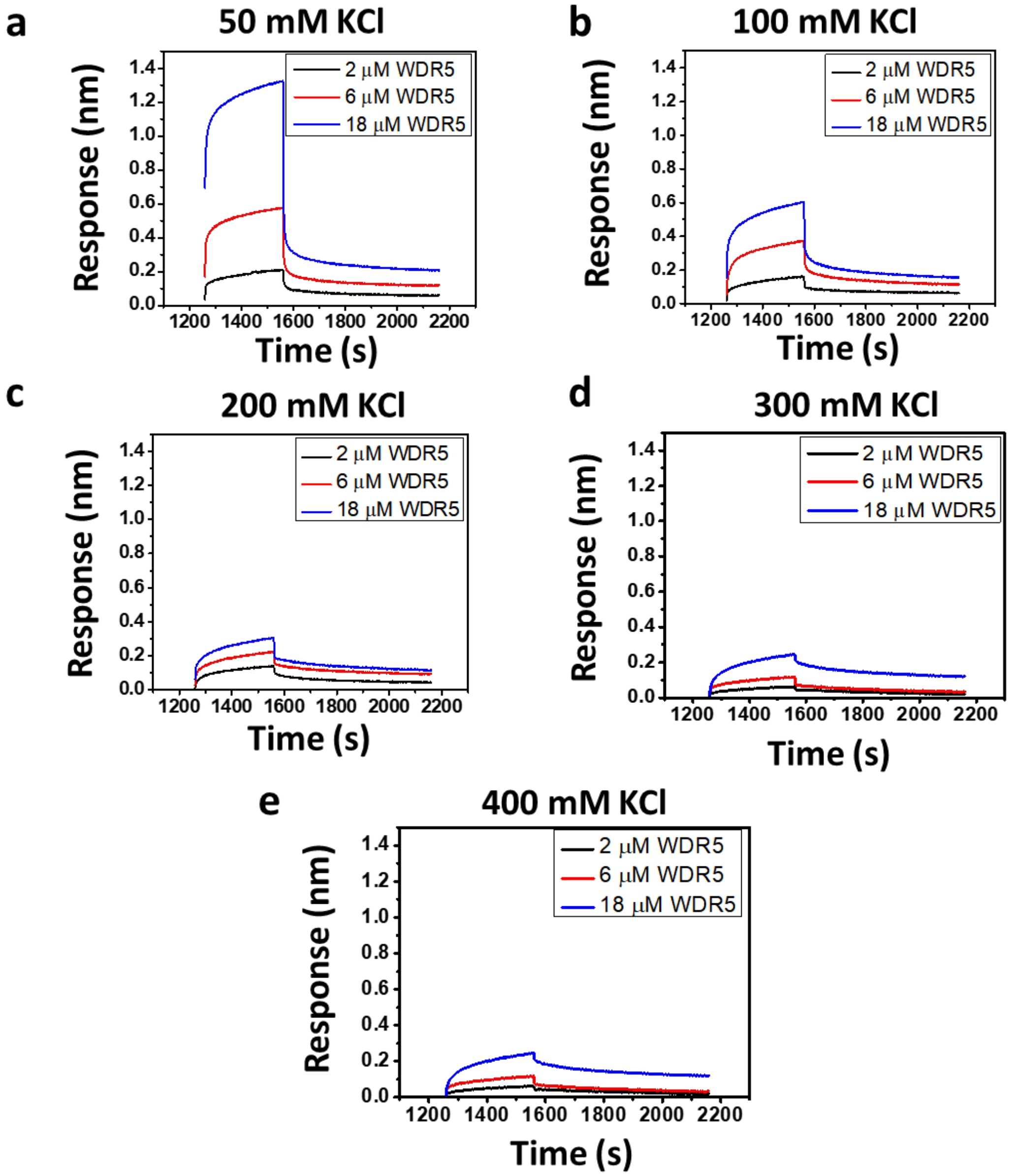

Figure S10. ND-BLI sensorgrams of the MYC$_{WBM}$tFhuA-WDR5 interaction at various [KCl] values. A serial dilution of WDR5 ranging from 2 μM to 18 μM was used to obtain individual binding curves. ND-BLI sensorgrams show a dose-dependent response following the interaction between MYC$_{WBM}$tFhuA and WDR5. MYC$_{WBM}$tFhuA was reconstituted into biotinylated ND to form the ND-MYC$_{WBM}$tFhuA complex (**Experimental section**). 15 nM biotin-tagged ND-MYC$_{WBM}$tFhuA was loaded onto streptavidin (SA) sensors for 5 minutes. **(a)** ND-BLI experiment executed in 50 mM KCl. **(b)** 100 mM KCl. **(c)** 200 mM KCl. **(d)** 300 mM KCl. **(e)** 400 mM KCl. These ND-BLI traces were replicated in $n = 3$ independent ND-BLI experiments. The other experimental conditions were the same as those stated in the **Experimental section**.

Table S14. Voltage dependence of the WDR5-unbound (τ_{on}) and WDR5-bound durations (τ_{off}) of the MYC$_{WBM}$-WDR5 interaction. τ_{on} are mean values of the single-exponential distributions of WDR5-unbound durations when 11.4 μM WDR5 was added to the *cis* side. τ_{off} values are the mean values of the single-exponential distributions of WDR5-bound durations. Δ*U* is the applied transmembrane potential. The maximum likelihood method[5-7] and logarithm likelihood ratio (LLR)[8-10, 21] tests were used for all fittings to determine the best model of the probability distribution function (PDF) of these time constants.

**Δ*U*** **(mV)**	**τ_{on}** **(s)**	**τ_{off}** **(s)**
-40	0.28 ± 0.04	0.044 ± 0.002
-20	0.71 ± 0.10	0.039 ± 0.003
-10	1.6 ± 0.6	0.037 ± 0.004
+10	3.4 ± 0.5	0.033 ± 0.002
+20	5.3 ± 0.9	0.031 ± 0.002
+40	11 ± 1	0.029 ± 0.002

Values are mean ± s.d. *n = 3,* where *n* is the number of independently reconstituted nanopores. The other experimental conditions were the same as those stated in the **Experimental section**.

Table S15. Voltage dependence of the association rate constants (k_{on}) and dissociation rate constants (k_{off}) for the MYC$_{WBM}$-WDR5 interaction. $k_{on}(0)$ represents the association rate constant in the absence of the applied transmembrane potential, Δ*U*. This value is the y-intercept of the linear fit in **Figure 5e.** τ_{on} values are provided in **Supplementary Table S14.** $k_{off}(0)$ represents the dissociation rate constant without an applied voltage. This value is the y-intercept of the linear fit in **Figure 5f.** τ_{off} values are provided in **Supplementary Table S14.**

n	**Δ*U*** **(mV)**	**k_{on}× 10^{-5} ($M^{-1}s^{-1}$)**	**$k_{on}(0)$ × 10^{-5} ($M^{-1}s^{-1}$)**	**k_{off} (s^{-1})**	**$k_{off}(0)$ (s^{-1})**
NA	from fitting	NA	0.48 ± 0.01	NA	28 ± 0.1
3	-40	3.1 ± 0.5		23 ± 1	
4	-20	1.3 ± 0.2		25 ± 1	
3	-10	0.6 ± 0.2		27 ± 2	
3	+10	0.3 ± 0.03		30 ± 1	
3	+20	0.2 ± 0.03		32 ± 2	
3	+40	0.08 ± 0.01		34 ± 2	

Values indicate mean ± s.d. *n* is the number of independently reconstituted nanopores. NA stands for not applicable data. $k_{on}(0)$ and $k_{off}(0)$ are provided as mean ± s.e.m. The other experimental conditions were the same as those stated in the **Experimental section**.

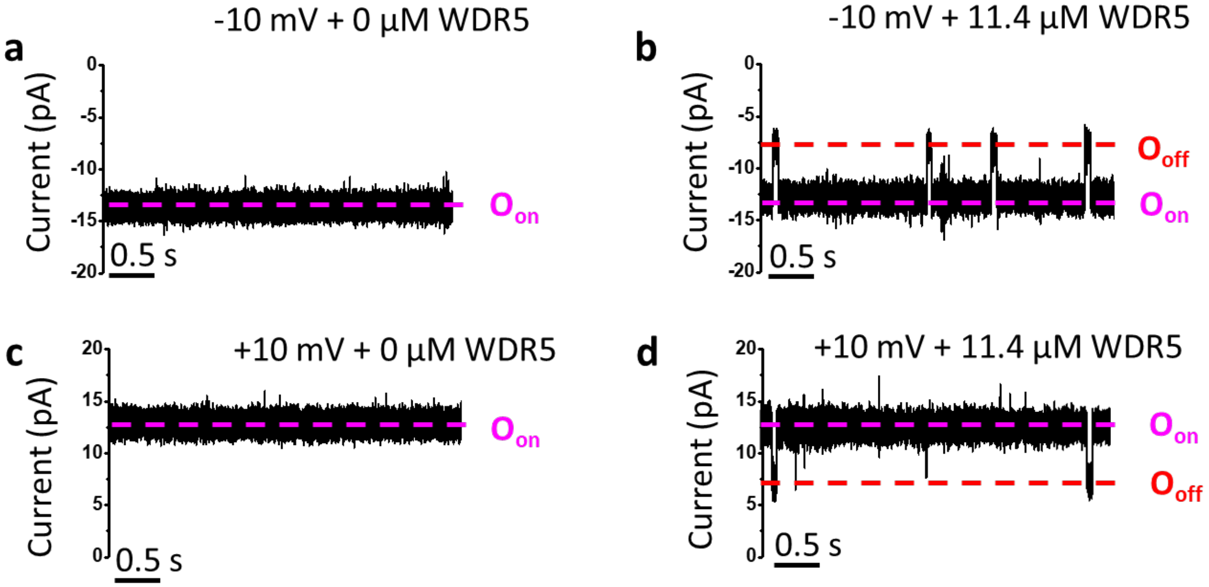

Figure S11. Representative single-channel electrical traces acquired in the absence and presence of WDR5 and at applied transmembrane potentials of +10 mV and -10 mV. These traces were low-pass filtered using an 8-pole Bessel filter at a frequency of 1 kHz. All recordings were conducted using a solution containing 300 mM KCl, 20 mM Tris-HCl, 1 mM TCEP, and pH 7.5. **(a)** A representative trace was acquired with MYC_{WBM}tFhuA without WDR5 and at +10 mV. **(b)** The same as (a) but in the presence of 11.4 μM WDR5. **(c)** The same as (a) but at -10 mV. **(d)** The same as (c) but in the presence of 11.4 μM WDR5. These traces were replicated in $n = 3$ independent single-molecule reconstitutions. The other experimental conditions were the same as those stated in the **Experimental section**.

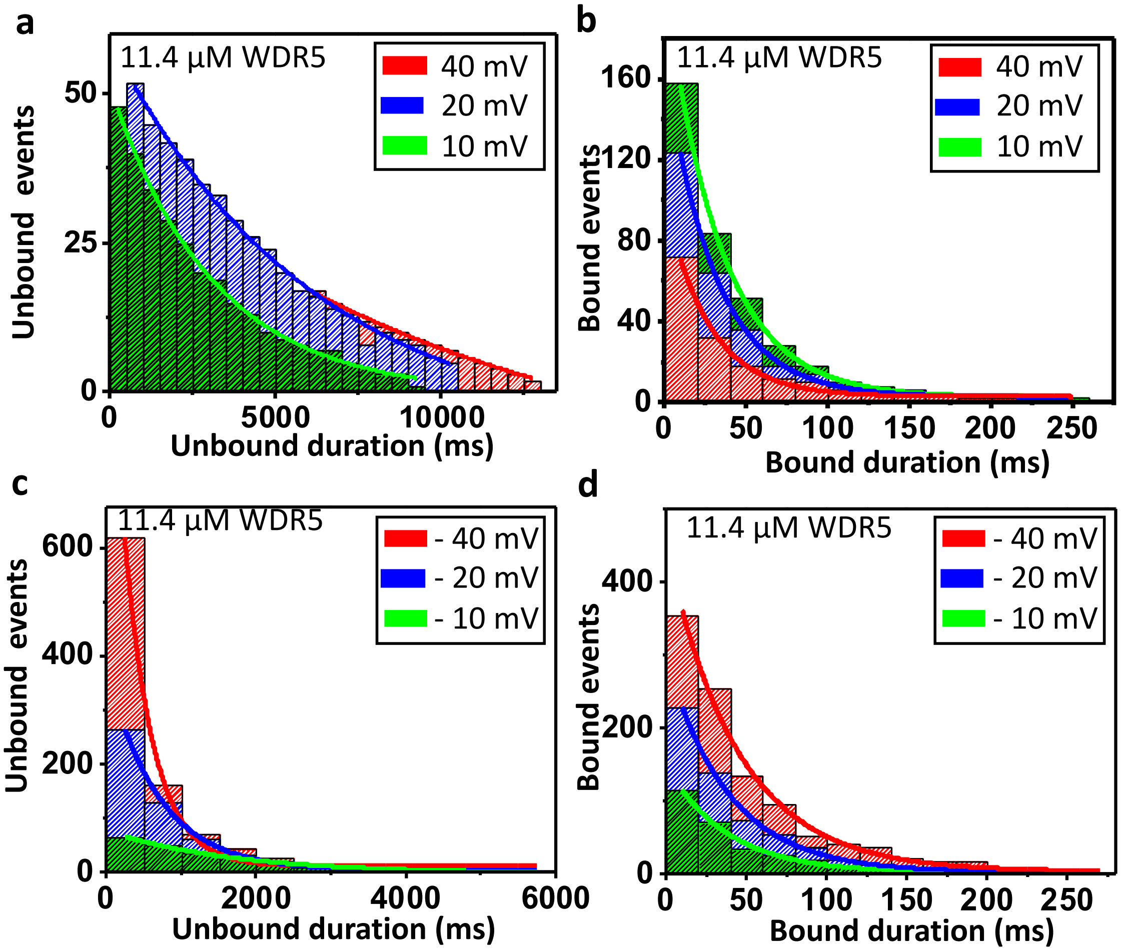

Figure S12. Duration histograms of the WDR5-unbound and WDR5-bound events at various applied transmembrane potentials. 11.4 µM WDR5 was added to the *cis* side. **(a)** Event histograms of the WDR5-unbound durations (τ_{on}). The τ_{on} values (mean ± s.e.m.) were 3140 ± 93 ms (number of events: N = 376), 6120 ± 570 ms (N =218), and 11020 ± 290 ms (N = 122) at +10, +20, and +40 mV, respectively. (**b**) Event histograms of the WDR5-bound durations (τ_{off}). The τ_{off} values (mean ± s.e.m.) were 33 ± 7.3 ms (number of events: N = 304), 30 ± 1.0 ms (N =286), and 28 ± 1.5 ms (N = 166) at +10, +20, and +40 mV, respectively. Data from (a) and (b) are taken from extended recordings of 25 minutes. **(c)** The same as (a) but for -10, -20, and -40 mV. The τ_{on} values (mean ± s.e.m.) were 380 ± 22 ms (number of events: N = 962), 690 ± 11 ms (N =544), and 1720 ±25 ms (N = 226) at -40, -20, and -10 mV, respectively. **(d)** The same as (b) but for -10, -20, and -40 mV, respectively. The τ_{off} values (mean ± s.e.m.) were 45 ± 2.9 ms (number of events: N = 1038), 39 ± 1.5 ms (N =594), and 38 ± 2.1 ms (N = 298) at -40, -20, and -10 mV, respectively. The other experimental conditions were the same as those stated in the **Experimental section**.

Table S16. The reduction in activation free energies of WDR5-unbound events, $\Delta\Delta G_{on}$, at different transmembrane potentials, ΔU, with respect to the value determined at a zero transmembrane potential. All values were calculated at 11.4 µM WDR5. The differential activation free energies, $\Delta\Delta G_{on}$, were calculated using the following equations:

$$\ln(k_{on}) = \ln(k_{on}(0)) - \frac{\Delta\Delta G_{on}}{RT}$$

R is the general gas constant. T denotes the absolute temperature. k_{on} (ΔU) and k_{on} (0) are provided in **Supplementary Table S15**.

n	**ΔU (mV)**	**$\Delta\Delta G_{on}$ (kcal/mol)**
3	–40	-1.1 ± 0.1
4	–20	-0.58 ± 0.08
3	–10	-0.14 ± 0.06
3	+10	0.34 ± 0.08
3	+20	0.60 ± 0.01
3	+40	1.0 ± 0.1

Values were provided as mean ± s.d. n is the number of independently reconstituted nanopores. The other experimental conditions were the same as those stated in the **Experimental section**.

Table S17. The relative charge of WDR5 is determined by voltage dependence experiments. All values were calculated at 11.4 µM WDR5. The association rate constant at a specific transmembrane potential, ΔU, is given by $k_{on}(\Delta U) = k_{on}(0)e^{-\frac{q\Delta U}{k_B T}}$. Here, $k_{on}(\Delta U)$ and $k_{on}(0)$ are the association rate constants at transmembrane potentials ΔU and 0 mV, respectively. Here, k_B is the Boltzmann constant, and T is the absolute temperature. q is the effective charge. The relative charge, z, of WDR5 was extracted using the slope of the linear fit of $k_{on}(\Delta U)$ in **Figure 5e**. The slope equals $-\frac{q}{k_B T}$, where $q = z$e. z and e denote the relative charge and elementary charge, respectively. k_{on} (ΔU) and k_{on} (0) are provided in **Supplementary Table S15**. The relative charge, z, has a positive value because the slope in **Figure 5e** is negative.

n	*z*
8	1.3 ± 0.2

The value is the mean ± s.e.m. n is the number of independently reconstituted nanopores. The other experimental conditions were the same as those stated in the **Experimental section**.

Table S18. The open-state currents of the MLL4$_{Win}$tFhuA biosensor. The open-state current, I_{on}, is directly measured at a transmembrane potential of -20 mV.

Nanopore Sensor	I_{on} (pA)
**MLL4$_{Win}$tFhuA**	-23 ± 1

This value is provided as mean ± s.d. obtained from $n = 4$ independently reconstituted nanopores for MLL4$_{Win}$tFhuA. The other experimental conditions were the same as those in the **Experimental section**.

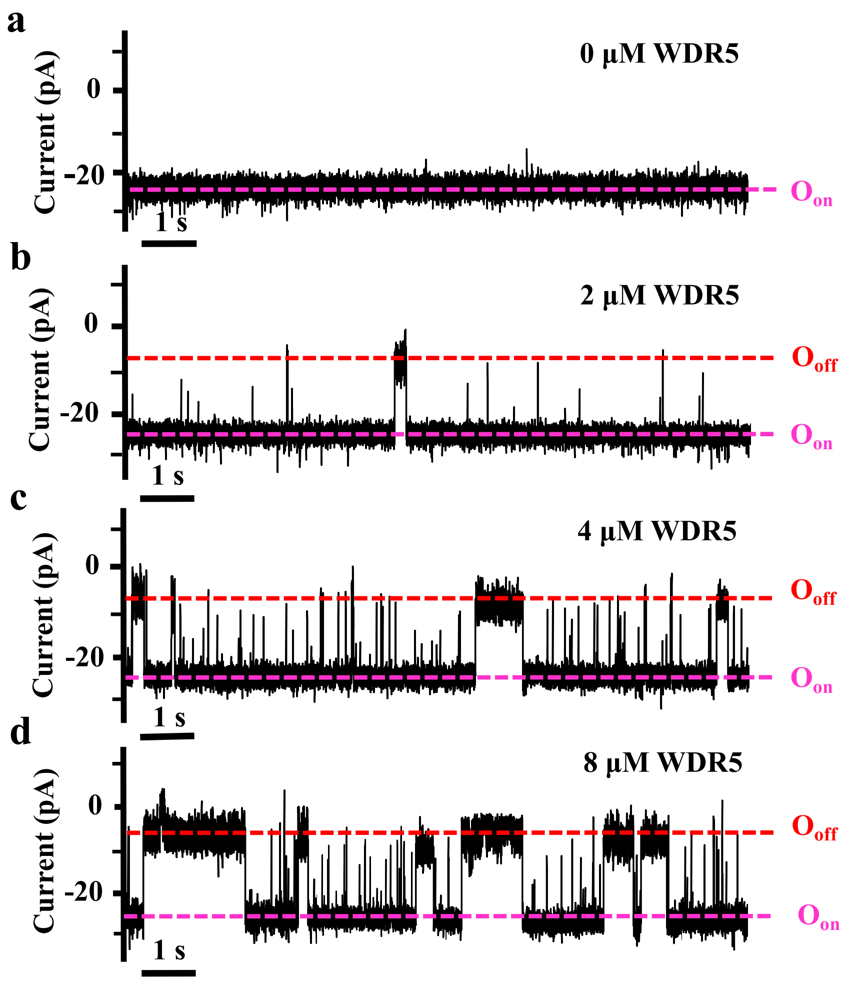

Figure S13. Representative single-channel electrical traces of MLL4$_{Win}$tFhuA when WDR5 was added to the *cis* compartment. These traces were low-pass Bessel filtered at a frequency of 0.1 kHz. They were performed in 300 mM KCl, 20 mM Tris-HCl, 1 mM TCEP, and pH 7.5. The applied transmembrane potential was -20 mV. **(a)** A representative single-channel electrical trace was recorded with a single MLL4$_{Win}$tFhuA at 0 µM WDR5. **(b)** The same trace as in (a), but in the presence of 2 µM WDR5 added to the *cis* side. **(c)** The same trace as in (a), but in the presence of 4 µM WDR5 added to the *cis* side. **(d)** The same trace as in (a), but in the presence of 8 µM WDR5 added to the *cis* side. O_{on} and O_{off} represent the WDR5-unbound and WDR5-bound substates, respectively. The other experimental conditions were the same as those in the **Experimental section**. This data was adapted from Mayse et al. (2022).[22]

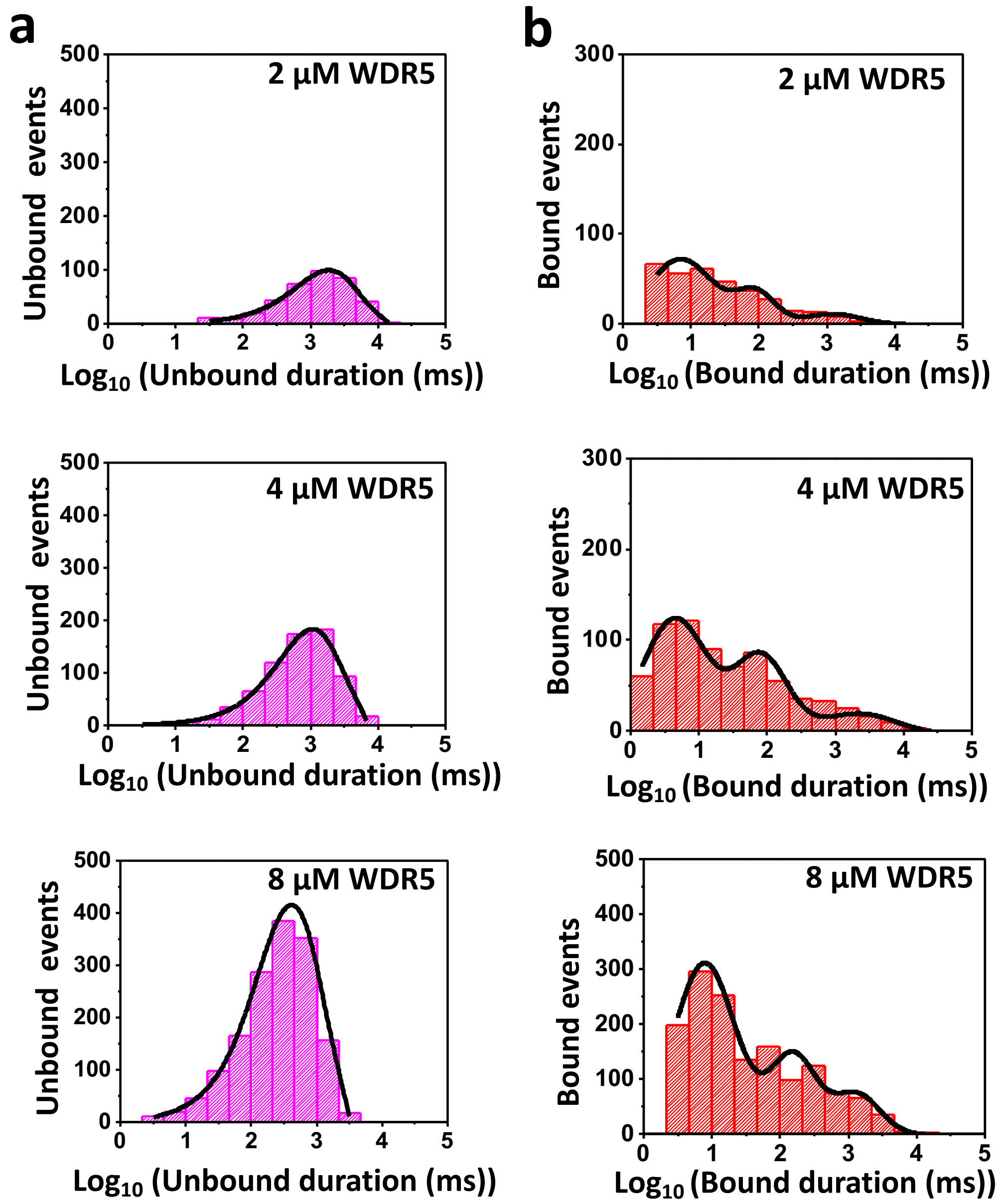

Figure S14. Duration histograms of the WDR5-unbound and WDR5-bound events recorded with the MLL4$_{Win}$tFhuA biosensor. (a) Histograms of WDR5-unbound event durations, whose τ_{on} values (mean ± s.e.m.) were 1.8 ± 0.4 s (number of events: N = 382), 1.5 ± 0.2 s (N = 709), and 0.51 ± 0.06 s (N = 1413) at 2, 4, and 8 µM WDR5, respectively. **(b)** Histograms of WDR5-bound event durations. The cumulative fits are marked in black. For 2 µM WDR5, these durations (mean ± s.e.m.) were 0.009 ± 0.001 s, 0.14 ± 0.002 s, and 1.9 ± 0.3 s, respectively (N = 329). For 4 µM WDR5, these durations (mean ± s.e.m.) were 0.009 ± 0.002 s, 0.11 ± 0.02 s, and 2.1 ± 0.1 s, respectively (N = 712). For 8 µM WDR5, these durations (mean ± s.e.m.) were 0.007 ± 0.002 s, 0.12 ± 0.02 s, and 1.7 ± 0.3 s, respectively (N = 1,398). This data is from the representative trace of a reconstituted nanopore in **Supplementary Figure S13**.

Table S19. The association and dissociation rate constants for $MLL4_{Win}$-WDR5 and MYC_{WBM}-WDR5 interactions using $MLL4_{Win}$tFhuA and MYC_{WBM}tFhuA nanopore sensors, respectively. $MLL4_{Win}$tFhuA data was adapted from Mayse et al. (2022).[22]

	$k_{on-1}\times 10^{-5}$ ($M^{-1}s^{-1}$)	$k_{on-2}\times 10^{-4}$ ($M^{-1}s^{-1}$)	$k_{on-3}\times 10^{-4}$ ($M^{-1}s^{-1}$)	k_{off-1} (s^{-1})	k_{off-2} (s^{-1})	k_{off-3} (s^{-1})	$k_{on}\times 10^{-5}$ ($M^{-1}s^{-1}$)	k_{off} (s^{-1})
MYC_{WBM}tFhuA							1.4 ± 0.1	26 ± 1
$MLL4_{Win}$tFhuA	1.4 ± 0.1	6.9 ± 1.8	3.6 ± 1.0	86 ± 2	9.2 ± 0.5	0.78 ± 0.06		

Values were provided as mean ± s.d. n = 8 for MYC_{WBM}tFhuA and n = 9 for $MLL4_{Win}$tFhuA. n is the number of independently reconstituted nanopores. The other experimental conditions were the same as those in the **Experimental section**.

Table S20. The equilibrium dissociation rate constants, K_D, for the $MLL4_{Win}$-WDR5 and MYC_{WBM}-WDR5 interactions using the $MLL4_{Win}$tFhuA and MYC_{WBM}tFhuA nanopore sensors, respectively. These K_D values correspond to measurements conducted in 300 mM KCl, 20 mM Tris-HCl, 1 mM TCEP, and pH 7.5. $MLL4_{Win}$tFhuA data was adapted from Mayse et al. (2022).[22]

Nanopore	K_D (μM)	K_{D-1} (μM)	K_{D-2} (μM)	K_{D-3} (μM)
MYC_{WBM}tFhuA	197 ± 2			
$MLL4_{Win}$tFhuA		631 ± 49	138 ± 18	20 ± 4

Values were provided as mean ± s.d. n = 8 for MYC_{WBM}tFhuA and n = 9 for $MLL4_{Win}$tFhuA. n is the number of independently reconstituted nanopores. The other experimental conditions were the same as those in the **Experimental section**.

Table S21. The mean normalized amplitudes (I/I_0) for $MLL4_{Win}$tFhuA and MYC_{WBM}tFhuA. I_0 and I denote the single-channel current of the WDR5-unbound substate and the amplitude of WDR5-bound current blockades of each sensor, respectively. 2 μM WDR5 was added to the *cis* side. $MLL4_{Win}$tFhuA revealed two distinct mean normalized amplitudes, $I/I_{0\text{-}1}$ and $I/I_{0\text{-}2}$. $MLL4_{Win}$tFhuA data was adapted from Mayse et al. (2022).[22]

[WDR5] (μM)	$MLL4_{Win}$tFhuA $I/I_{0\text{-}1}$	$MLL4_{Win}$tFhuA $I/I_{0\text{-}2}$	MYC_{WBM}tFhuA I/I_0
2	61 ± 3	72 ± 2	41 ± 3
4	60 ± 1	69 ± 2	40 ± 2
7.8/8	59 ± 4	72 ± 3	41 ± 4

Values indicate mean ± s.d. n is the number of independently reconstituted nanopores. n = 8 for MYC_{WBM}tFhuA and n = 9 for $MLL4_{Win}$tFhuA. 7.8 μM WDR5 was added to the *cis* side in experiments with MYC_{WBM}tFhuA. 8 μM WDR5 was added to the *cis* side in experiments with $MLL4_{Win}$tFhuA. The other experimental conditions were the same as those in the **Experimental section**.

Table S22. The relative charges of WDR5 were determined by voltage dependence experiments with $MLL4_{Win}$tFhuA and MYC_{WBM}tFhuA. The relative charge (z) from MYC_{WBM}tFhuA is provided in **Supplementary Table S17**. The charges for $MLL4_{Win}$tFhuA were extracted from Mayse et al. (2022).[22]

MYC_{WBM}tFhuA z	$MLL4_{Win}$tFhuA z_{-1}	$MLL4_{Win}$tFhuA z_{-2}	$MLL4_{Win}$tFhuA z_{-3}
1.25 ± 0.2	0.81 ± 0.07	0.76 ± 0.10	0.79 ± 0.12

Values were provided as mean ± s.e.m. n = 8 for MYC_{WBM}tFhuA and n = 9 for $MLL4_{Win}$tFhuA. Here, n is the number of independently reconstituted nanopores. The other experimental conditions were the same as those in the **Experimental section.**

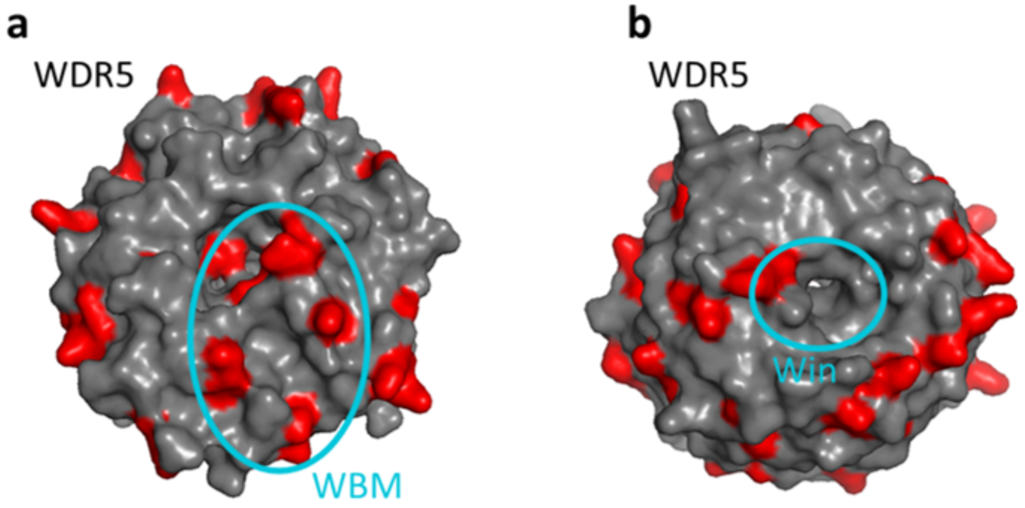

Figure S15. Positive charge regions of WDR5 at the WBM and Win sites. A cartoon of WDR5 (gray) with all its positively charged amino acids labeled in red. **(a)** The approximate WBM site and surrounding residues are circled in cyan. (**b**) The approximate Win site cavity is circled in cyan.

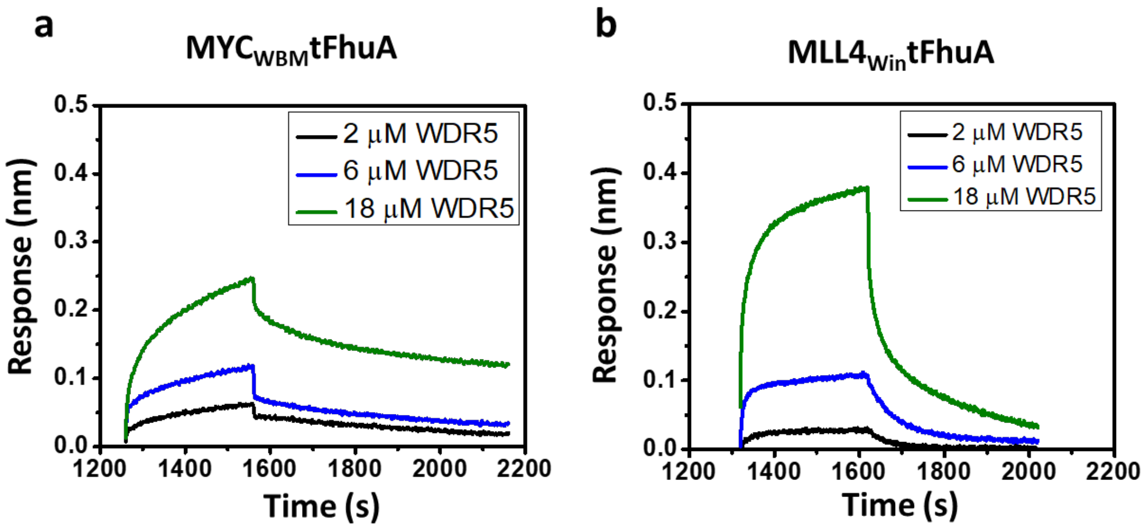

Figure S16. BLI sensorgrams of the MYC$_{WBM}$tFhuA and MLL4$_{Win}$tFhuA nanopore sensors interacting with WDR5. A serial dilution of WDR5 ranging from 2 μM to 18 μM was used to obtain individual binding curves. MYC$_{WBM}$tFhuA and MLL4$_{Win}$tFhuA were reconstituted into biotinylated nanodisc (ND) as mentioned in the **Experimental section** to form ND-MYC$_{WBM}$tFhuA and ND-MLL4$_{Win}$tFhuA complexes. 15 nM biotin-tagged ND-MYC$_{WBM}$tFhuA and ND-MLL4$_{Win}$tFhuA were loaded onto streptavidin-coated sensors for 5 minutes. **(a)** ND-BLI sensorgrams that show a dose-dependent response following the MYC$_{WBM}$-WDR5 interaction. **(b)** The same as (a) but for the MLL4$_{Win}$-WDR5 interaction probed with MLL4$_{Win}$tFhuA. The MLL4$_{Win}$tFhuA data was adapted from Mayse et al. (2023).[11]

Table S23. ND-BLI-determined kinetic rate constants for the binding of WDR5 with MLL4$_{Win}$ and MYC$_{WBM}$ using the MLL4$_{Win}$tFhuA and MYC$_{WBM}$tFhuA nanopores, respectively. Data acquired with MLL4$_{Win}$tFhuA was adapted from Mayse et al. (2023).[11] The ND-BLI-determined equilibrium dissociation constant, K_D, of the MLL4$_{Win}$-WDR5 interaction was ~25.8 μM.

	$k_{on} \times 10^{-4}$ ($M^{-1}s^{-1}$)	k_{off} (s^{-1})
**MLL4$_{Win}$tFhuA**	1.9 ± 0.2	0.46 ± 0.02
**MYC$_{WBM}$tFhuA**	NA	NA

The values indicate the mean ± s.d. $n = 3$ independently executed ND-BLI experiments. NA stands for not available because ND-BLI data could not be employed for a satisfactory global fit. The other experimental conditions were the same as those in the **Experimental section**.

REFERENCES

1. Tina, K. G.; Bhadra, R.; Srinivasan, N., PIC: Protein Interactions Calculator. *Nucleic Acids Res.* **2007,** *35* (Web Server issue), W473-6.

2. Thomas, L. R.; Wang, Q.; Grieb, B. C.; Phan, J.; Foshage, A. M.; Sun, Q.; Olejniczak, E. T.; Clark, T.; Dey, S.; Lorey, S.; Alicie, B.; Howard, G. C.; Cawthon, B.; Ess, K. C.; Eischen, C. M.; Zhao, Z.; Fesik, S. W.; Tansey, W. P., Interaction with WDR5 promotes target gene recognition and tumorigenesis by MYC. *Mol. Cell. Biochem.* **2015,** *58* (3), 440-52.

3. Thakur, A. K.; Movileanu, L., Real-Time Measurement of Protein-Protein Interactions at Single-Molecule Resolution using a Biological Nanopore. *Nature Biotechnol.* **2019,** *37* (1), 96-101.

4. Thakur, A. K.; Movileanu, L., Single-Molecule Protein Detection in a Biofluid Using a Quantitative Nanopore Sensor. *ACS Sens.* **2019,** *4* (9), 2320-2326.

5. Colquhoun, D.; Hatton, C. J.; Hawkes, A. G., The quality of maximum likelihood estimates of ion channel rate constants. *J. Physiol.* **2003,** *547* (Pt 3), 699-728.

6. Hatton, C. J.; Shelley, C.; Brydson, M.; Beeson, D.; Colquhoun, D., Properties of the human muscle nicotinic receptor, and of the slow-channel myasthenic syndrome mutant epsilonL221F, inferred from maximum likelihood fits. *J Physiol* **2003,** *547* (Pt 3), 729-60.

7. Colquhoun, D.; Sigworth, F. J., Fitting and statistical analysis of single-channel records. In *Single-channel recording*, 2nd ed.; Sackmann, B., Neher,E., Ed. Plenum Press: New York, 1995; pp 483-587.

8. McManus, O. B.; Blatz, A. L.; Magleby, K. L., Sampling, Log Binning, Fitting, and Plotting Durations of Open and Shut Intervals From Single Channels and the Effects of Noise. *Pflugers Arch.* **1987,** *410* (4-5), 530-553.

9. McManus, O. B.; Magleby, K. L., Kinetic States and Modes of Single Large-Conductance Calcium-Activated Potassium Channels in Cultured Rat Skeletal-Muscle. *J. Physiol. (Lond.)* **1988,** *402*, 79-120.

10. Movileanu, L.; Cheley, S.; Bayley, H., Partitioning of Individual Flexible Polymers into a Nanoscopic Protein Pore. *Biophys. J.* **2003,** *85* (2), 897-910.

11. Mayse, L. A.; Imran, A.; Wang, Y.; Ahmad, M.; Oot, R. A.; Wilkens, S.; Movileanu, L., Evaluation of Nanopore Sensor Design Using Electrical and Optical Analyses. *ACS Nano* **2023,** *17* (11), 10857-10871.

12. Wolfe, A. J.; Si, W.; Zhang, Z.; Blanden, A. R.; Hsueh, Y. C.; Gugel, J. F.; Pham, B.; Chen, M.; Loh, S. N.; Rozovsky, S.; Aksimentiev, A.; Movileanu, L., Quantification of membrane protein-detergent complex interactions. *J. Phys. Chem. B* **2017,** *121* (44), 10228-10241.

13. Wolfe, A. J.; Hsueh, Y. C.; Blanden, A. R.; Mohammad, M. M.; Pham, B.; Thakur, A. K.; Loh, S. N.; Chen, M.; Movileanu, L., Interrogating Detergent Desolvation of Nanopore-Forming Proteins by Fluorescence Polarization Spectroscopy. *Anal. Chem.* **2017,** *89* (15), 8013-8020.

14. Smith, A. M.; Lee, A. A.; Perkin, S., The Electrostatic Screening Length in Concentrated Electrolytes Increases with Concentration. *J. Phys. Chem. Lett.* **2016,** *7* (12), 2157-63.

15. Debye, P.; Hückel, E., Zur Theorie der Elektrolyte. . *Phys. Z.* **1923** *24* 185–206.

16. Robinson, R. A.; Stokes, R. H., *Electrolyte Solutions* Butterworths: London, 1959.

17. Schreiber, G.; Fersht, A. R., Rapid, electrostatically assisted association of proteins. *Nat. Struct. Biol.* **1996,** *3* (5), 427-31.

18. Manov, G. G.; Bates, R. G.; Hamer, W. J.; Acree, S. F., Values of the constants in the Debye-Hückel equation for activity coefficients. . *J. Am. Chem. Soc.* **1943** *65* (9), 1765–1767.

19. Kielland, J., Individual activity coefficients of ions in aqueous solutions. . *J. Am. Chem. Soc.* **1937** *59* (9), 1675–1678.

20. Harrington, L.; Cheley, S.; Alexander, L. T.; Knapp, S.; Bayley, H., Stochastic detection of Pim protein kinases reveals electrostatically enhanced association of a peptide substrate. *Proc. Natl. Acad. Sci. U.S.A* **2013,** *110* (47), E4417-E4426.

21. Couoh-Cardel, S.; Hsueh, Y. C.; Wilkens, S.; Movileanu, L., Yeast V-ATPase Proteolipid Ring Acts as a Large-conductance Transmembrane Protein Pore. *Sci. Rep.* **2016,** *6*, 24774.

22. Mayse, L. A.; Imran, A.; Larimi, M. G.; Cosgrove, M. S.; Wolfe, A. J.; Movileanu, L., Disentangling the recognition complexity of a protein hub using a nanopore. *Nature Commun.* **2022,** *13* (1), 978.
